# Evolutionary origin of peptidoglycan recognition proteins in vertebrate innate immune system

**DOI:** 10.1186/1471-2148-11-79

**Published:** 2011-03-25

**Authors:** Adriana M Montaño, Fumi Tsujino, Naoyuki Takahata, Yoko Satta

**Affiliations:** 1 Department of Biosystems Science, School of Advanced Sciences, The Graduate University for Advanced Studies (Sokendai), Shonan Village, Hayama, 240-0193, Japan; 2 Department of Pediatrics, School of Medicine, Saint Louis University, 1100 S. Grand Blvd, Doisy Research Center, Saint Louis, MO, 63104, USA

## Abstract

**Background:**

Innate immunity is the ancient defense system of multicellular organisms against microbial infection. The basis of this first line of defense resides in the recognition of unique motifs conserved in microorganisms, and absent in the host. Peptidoglycans, structural components of bacterial cell walls, are recognized by Peptidoglycan Recognition Proteins (PGRPs). PGRPs are present in both vertebrates and invertebrates. Although some evidence for similarities and differences in function and structure between them has been found, their evolutionary history and phylogenetic relationship have remained unclear. Such studies have been severely hampered by the great extent of sequence divergence among vertebrate and invertebrate PGRPs. Here we investigate the birth and death processes of PGRPs to elucidate their origin and diversity.

**Results:**

We found that (i) four rounds of gene duplication and a single domain duplication have generated the major variety of present vertebrate PGRPs, while in invertebrates more than ten times the number of duplications are required to explain the repertoire of present PGRPs, and (ii) the death of genes in vertebrates appears to be almost null whereas in invertebrates it is frequent.

**Conclusion:**

These results suggest that the emergence of new *PGRP *genes may have an impact on the availability of the repertoire and its function against pathogens. These striking differences in PGRP evolution of vertebrates and invertebrates should reflect the differences in the role of their innate immunity. Insights on the origin of *PGRP *genes will pave the way to understand the evolution of the interaction between host and pathogens and to lead to the development of new treatments for immune diseases that involve proteins related to the recognition of self and non-self.

## Background

Innate immunity is the ancient defense system of multicellular organisms against microbial infection. The basis of this first line of defense resides in the recognition of unique motifs or components conserved in microorganisms, and absent in the host. The innate immune system uses sets of pattern recognition receptors to recognize such foreign or non-self motifs. Proteins in the immune system can be located intracellularly, on the cell surface, or secreted into the bloodstream, ready to signal the presence of an intruder in every compartment. In systems lacking the adaptive arm of immunity, the pattern recognition concept serves well to explain the general triggering of the system as well as providing receptors for the limited specificity shown by innate immunity [1-4].

Peptidoglycan (PGN) is the major structural component of the cell wall of almost all bacterial species. PGN is a large, repetitive macromolecule that forms the rigid cell wall of bacteria. PGN recognition is mediated by the PGRP (PGN recognition protein) family of receptors [5,6]. PGRPs are a family of innate immunity pattern recognition molecules that were first discovered in silkworms [7]. There are four loci for each PGRPs in humans [8,9] (*PGRP-S, PGRP-L, PGRP-Iα *and *PGRP-Iβ*), while thirteen loci in *Drosophila *[10,11], which encode approximately 17 PGRP proteins through alternative splicing, and seven loci in *Anopheles *[12]. Several other genomes also show relatively large number of PGRPs in invertebrates, but only up to five in vertebrates (Additional file 1). In invertebrates, the functional divergence of each PGRP molecule is well investigated: Some possess an amidase activity that hydrolyzes the amide bond between the N-acetylmuramic acid and the L-alanine of peptidoglycan, others activate *Toll*, or *Imd *pathways to induce an expression of anti-bacteria peptides, induce prophenoloxidase cascade, or directly cause phagocytosis and lysis [13-18]. On the other hand, functions of vertebrate, or mainly mammalian PGRPs, are not fully understood [19]. While PGRP-L has the amidase activity where its role could be to detoxify PGN fragments present in blood and modulate the immune response as insect PGRPs, the PGRP-S, PGRP-Iα and PGRP-Iβ have bacteriostatic and/or bactericidal function [6,20-22].

Vertebrates have the acquired immunity system in addition to the innate immunity system, while in insects only the latter is a self-defense system. It is of interest whether possessing acquired immunity has effects on the evolution of molecules involved in innate immunity. Here, we investigate the birth and death processes of *PGRP*s by systematically analyzing *PGRP *genes from a set of diverse eukaryotes, and discuss the role of selection and diversification of this gene family.

## Results

### Modes of PGRP evolution

To detect lineage-specific expansions 40 sequences of the PGRP family from 21 vertebrate species and 42 sequences from 6 invertebrate species were studied (Additional file 1 and Additional file 2). Both vertebrate and invertebrate PGRPs have a highly conserved C-terminal region of the PGRP domain with three sub-domains (I, II and III). The sub-domains are determined by sequence conservation and not by their function. The PGRP domain shows a sequence similarity (~35%) with bacteriophage T7 lysozyme, which also has amidase activity, indicating that T7 lysozyme would be the origin of PGRP domains [23]. This ancient origin of PGRP domains is also supported by the similarity of the 3D structure between PGRP-L and T7 lysozyme molecules. However, the orthologous relationship of vertebrate and insect PGRP domains has not been ensured [8,19] due to the limited number of amino acid sites compared and the great extent of sequence divergence among them. Contrary to the conserved C-terminal PGRP domains, the N-terminal region shows no particular similarities among different PGRPs in invertebrates, and partial similarities among PGRP-S, PGRP-Iα and PGRP-Iβ in vertebrates. Therefore, we used only the PGRP domains for the alignment and tree construction. Due to difficulties in identifying orthologous relationship, we performed phylogenetic analyses of *PGRP *genes in vertebrates and invertebrates separately by using the neighbor-joining (NJ) and minimum evolution (ME) methods. There was no conflict in the topologies obtained with these methods.

**Figure 1:**
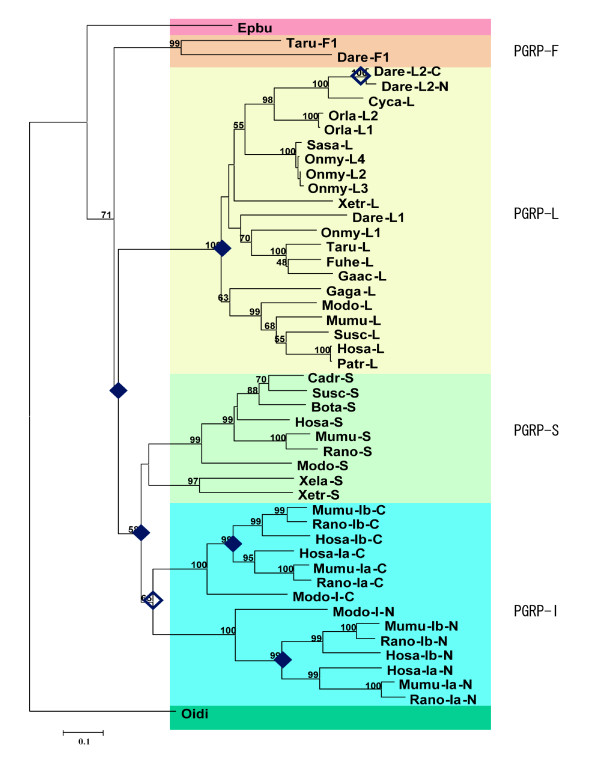
**Neighbor-joining tree of vertebrate PGRP amino acid sequences**. Filled and open diamonds indicate duplication of loci and domains, respectively. The analyzed sequences contain 145 amino acid sites. Numbers at the nodes represent the bootstrap support for the branch based on 1000 replications. The evolutionary distances were computed using the Poisson correction method and are in the units of the number of amino acid substitutions per site. All positions containing gaps and missing data were eliminated from the dataset. Notation of species names are indicated as follow: Bota (*Bos taurus*), Cadr (*Camelus dromedarius*), Cyca (*Cyprinus carpio*), Dare (*Danio renio*), Epbu (*Eptatretus burgeri*), Fuhe (*Fundulus heteroclitus*), Gaac (*Gasterosteus aculeatus*), Gaga (*Gallus gallus*), Hosa (*Homo sapiens*), Modo (*Monodelphis domestica*), Mumu (*Mus musculus*), Oidi (*Oikopleura dioica*), Onmy (*Oncorhynchus mykiss*), Orla (*Oryzias latipes*), Patr (*Pan troglodytes*), Rano (*Rattus norvegicus*), Sasa (*Salmo salar*), Susc (*Sus scrofa*), Taru (*Takifugu rubripes*), Xela (*Xenopus laevis*) and Xetr (*Xenopus tropicalis*).

In vertebrate PGRPs, the phylogeny shows five clustering groups, four of which corresponds to four loci found in humans; *PGRP-L*, *PGRP-S*, *PGRP-I*α and *PGRP-I*β. On the other hand, the fifth locus, named *PGRP-F*, is found only in fish. Including *PGRP-F*, there are four rounds of gene duplication and a single round of domain duplication, which produced the present-day vertebrate *PGRP*s (Figure 1, Additional file 3). The first round of gene duplication happened in the stem lineage leading to all jawed vertebrates. This duplication produced *PGRP-F *and the proto-*PGRP *that is an ancestor of *PGRPs *in other jawed vertebrates. In the second round, gene duplication occurred just after the first round and produced proto-*PGRP-L *and proto-*PGRP-S*. In addition to these two rounds, there is at least, an additional duplication in proto-*PGRP-L *in the lineage leading to fish *PGRP-L*. On the other hand, no descendant of proto-*PGRP-S *was detected in fish genomes. *PGRP-S *and proto-*PGRP-I *were produced after one round of duplication in proto-*PGRP-S *descendant in the stem lineage leading to tetrapods. The presence of *PGRP-S *in amphibians suggests the loss of proto-*PGRP-I *in this lineage. Just after this duplication and before the divergence of therian mammals, the proto-*PGRP-I *possesses two PGRP domains [24] due to the domain duplication 252~336 million years ago. After the divergence of opossums from placental mammals, the last round of gene duplication occurred 126~168 MYA producing *PGRP-I*α and *PGRP-I*β. This observation indicates that *PGRP-I*α and *PGRP-I*β are placental mammal-specific genes.

**Figure 2:**
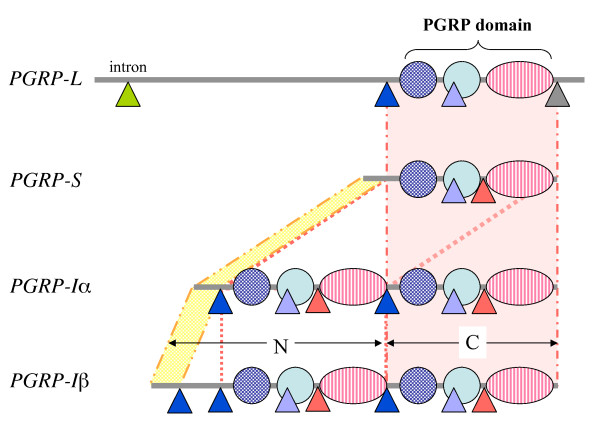
**Gene structure of four human *PGRP *genes**. Vertical lines indicate corresponding regions between different genes. A triangle shows the position of the introns and the same colour indicates that the position was shared.

The gene structure of vertebrate *PGRPs *(Figure 2) supports the above scenario, which explains the emergence of the vertebrate *PGRP*s. The *PGRP-S *contains two introns, one of which shares the position with both *PGRP-I *and *PGRP-L*, while the other only with *PGRP-I*. The position of this intron is preserved in the duplicated PGRP domains of *PGRP-I*. Further, the N-terminal regions of *PGRP-I*α and -*I*β genes show some sequence similarity with the *PGRP-S *N-terminal amino acid sequence. These observations indicate that the *PGRP-L *diverged first, *PGRP-I *is originated from *PGRP-S*, and the second PGRP domain in *PGRP-I *has been produced by domain duplication in *PGRP-S*. In addition to the main events, which originated the PGRP family commonly found in mammals, we also observed a recent domain duplication event in zebrafish *PGRP-L *where the domains exhibit homology of 99% (Figure 1).

**Figure 3:**
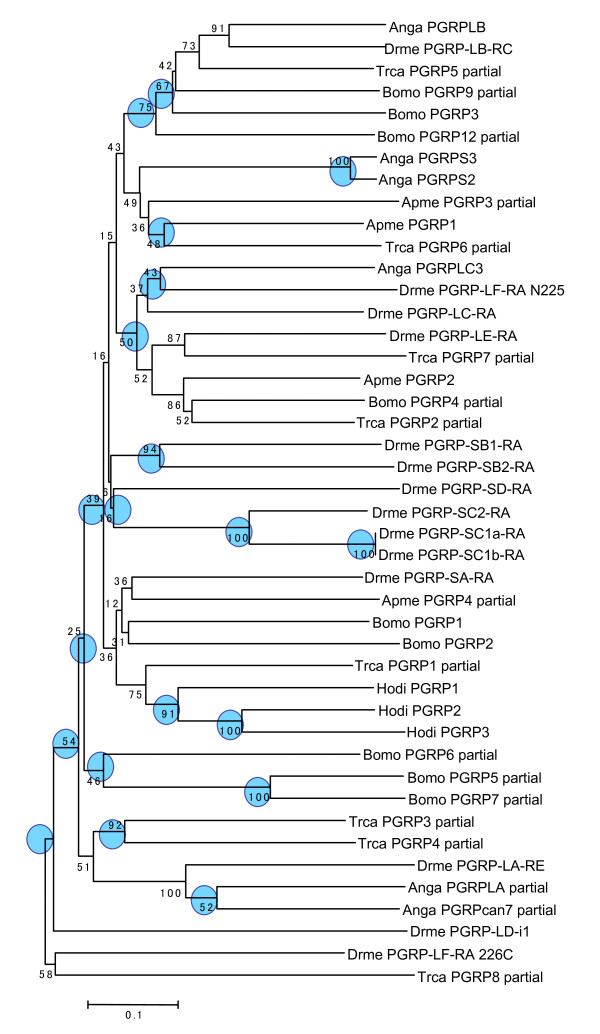
**Neighbor-joining tree of invertebrate PGRP amino acid sequences**. Circles on nodes indicate orthologous pairs of genes. The analysed sequences contain 207 amino acid sites. The evolutionary distances were computed using the Poisson correction method and are in the units of the number of amino acid substitutions per site. All positions containing gaps and missing data were eliminated from the dataset. Notation of species names are indicated as follows: Anga (*Anopheles gambiae*), Apme (*Apis mellifera*), Bomo (*Bombyx mori*), Drme (*Drosophila melanogaster*), Hodi (*Holotrichia diomphalia*), and Trca (*Tribolium castaneum*).

In contrast to this relatively small number of gene duplications and gene losses in vertebrate *PGRPs*, the birth and death process shows a different pattern in invertebrate *PGRP*s. The number of *PGRP *loci in the invertebrate genome ranges from four in *A. mellifera *to 14 in *B. mori*. Using 44 different sequences retrieved from databases (Additional file 2), we reconstructed the phylogenetic tree of PGRP domains for insects. In contrast to vertebrates, invertebrate *PGRP *genes are not clearly classified into orthologous groups. As representatives of the class Insecta, we used four genomes, which correspond to four different orders (*Diptera, Hemiptera, Coleoptera*, and *Lepidoptera*). The divergence time of these orders is similar to that of vertebrates. Gene duplication and loss are rather frequent and taxon-specific sets of *PGRP*s are evident. For example, in *Drosophila *only six of the thirteen are found in different orders, and seven seem to be *Drosophila *specific. The phylogenetic tree reveals that at least 14 rounds of gene duplication and two of gene losses are required to produce the extant repertoire of *PGRPs *in *Drosophila *genome (Figure 3, Additional file 4). A similar pattern of species-specific gene duplication and gene loss is observed in other insects, too.

The mentioned observation could be confirmed by using other methods that can predict the number of gene gains and losses. We verified our observations by using the program NOTUNG and EvolMAP [25,26]. The results with NOTUNG showed a similar tendency of the number of gene gains predicted (Additional file 5 and Additional file 6), however the number of gene losses seems to be overestimated in vertebrates, especially in fish. In NOTUNG, absence of a gene in a particular taxon means gene loss. Thus the number of losses in fish became enormously large. EvolMAP, on the other hand, predicts that gene gains are 8.6 times more frequent than gene losses in invertebrates over all branches. This suggests an expansion of *PGRPs *in invertebrates. For vertebrates, EvolMAP analysis shows no evidence of expansion or contraction for this gene family (Additional file 7 and Additional file 8). Overall, for the genes and species analyzed here, we find that the number of gains detected in invertebrates is twice the number of gains in vertebrates. Thus we could confirm the large number of gene gains and losses in invertebrates when compared to vertebrates.

### Ancestral PGRP genes

Due to the limited number of sites compared and long divergence time of sequences, we could not elucidate the relationship among the ancestors of vertebrate and invertebrate PGRPs from the phylogenetic tree of vertebrate and invertebrate PGRPs, including T7 lysozyme. Thus whether the origin of vertebrate PGRPs is monophyletic or paraphyletic to invertebrate PGRPs remains to be an open problem. However, our analysis clearly shows that for vertebrate PGRPs, the first major divergence took place between PGRP-L and PGRP-S. Therefore, in the following we focus on the vertebrate PGRPs to infer the functions of the ancestral *PGRP *genes.

To elucidate the function of ancestral PGRP molecules in vertebrates, the amino acid sequences of proto- *PGRP-L *and *PGRP-S *molecules were estimated by the maximum-likelihood (ML) method with the JTT substitution matrix [27]. It is known that seven amino acids are responsible for PGRP function [1,8]. Four amino acid residues (H17, Y46, H122, and C130) are essential for the amidase activity, whereas three (H36, W41, and K128) are important for Zn^2+ ^ligand-binding in the bacteriophage T7 lysozyme. Since all seven amino acids are conserved in both the proto- *PGRP-L *and *PGRP-S *sequences, the ancestor of both *proto-PGRPs *is likely to possess the amidase activity (Additional file 9). While the present-day *PGRP-L *has reserved its original function of amidase activity [20], *PGRP-S *has lost it and instead obtained the bacteriostatic function [21,22]. On the other hand, the invertebrate PGRPs possessing the amidase activity are paraphyletic to each other. This suggests independent gain or loss of the amidase activity in invertebrate *PGRP *at an early stage of the evolution.

### Selection acting on PGRPs and Evolutionary Rates

Next important question is whether some kind of selection process has acted on each amino acid site of vertebrate *PGRP *genes that will lead to their functional divergence after gene duplication. We identified positively or negatively selected sites in vertebrate *PGRP*s using Single Likelihood Ancestor Counting (SLAC) analysis as described in Methods [28]. Only the site 138 in *PGRP-L *is positively selected among all the vertebrate *PGRPs*. This site, which is involved in substrate binding, shows a high degree of amino acid variation in *PGRP-L *of different species.

Average values of the ratios of non-synonymous to synonymous substitutions of *PGRP-S*, *L *and *I *are 0.16, 0.13 and 0.17, respectively, and the overall value for *PGRP*s is 0.20. This indicates strong functional constraint, suggesting that amino acid sequences of these domains are well conserved among vertebrates.

Although there is a report where a few positively selected sites were observed in the PGRP domain of *Drosophila PGRP-LC *[29], we could not apply the above SLAC analysis to invertebrate *PGRPs*, because the clustering pattern and phylogenetic relationship among sequences are not ensured and the results of SLAC strongly depend on the tree topology (data not shown).

**Figure 4:**
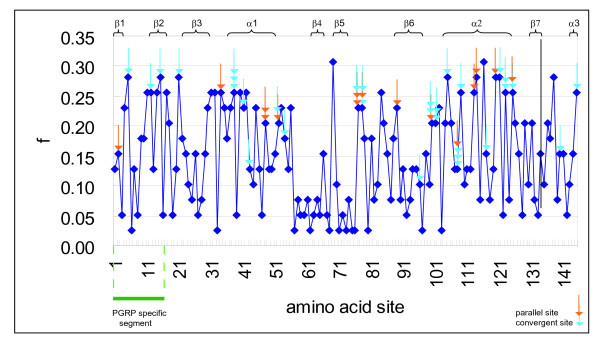
**Profile of the variation of amino acid sites, and occurrence of convergent and parallel substitutions**. The PGRP specific segment absent in T7 lysozyme is indicated. Orange arrows correspond to parallel substitutions, and blue arrows indicate convergent substitutions. The secondary structure assignment is depicted above the profile. *f *is defined as the ratio of the number of different amino acids at a specified site to the total number of sequences compared.

We further examined parallel and convergent evolution at the amino acid level to infer the operation of natural selection. We aligned 39 vertebrate PGRP sequences for each locus and deduced the ancestral amino acids [27] at all internal nodes of the phylogenetic tree (Figure 1), in order to estimate the presence of parallel and convergent substitutions, which may have been driven by the functional importance of sites. Subsequently the probability of a parallel or convergent substitution by chance was estimated as described in Methods. Our analysis revealed that thirteen sites have experienced parallel and twenty-three sites convergent substitutions, of which occurrence is statistically significant (p ≤ 0.05) (Table 1, Figure 4). Comparison of these sites based on the tertiary structure of *Drosophila *PGRP-LB [23], *Drosophila *PGRP-SA [30], and Human PGRP-Iα [24] showed that all these residues are located on α helices (sites 38, 41, 43, 45 and 52 in α1; 105, 108, 109, 113, 114, 117, 120, 121 and 123 in α 2; and 145 in α 3) and on β-sheets (site 2 on β1; 12 and 15 on β2; 89 and 96 on β6). Sites 2, 5, 12 and 15 are located on the PGRP specific fragment (Table 1).

**Table 1:** Tests of parallel and convergent evolution of PGRPs.

Species	n	P	Site positions
			
**A. Parallel changes**			
			
Pig-S & Cow-S	3	0.003	2, 78, 113
Frog-S2 & Rat-S	2	0.02*	34, 77
Rainbow trout-L1 & stickleback-L	3	0.006	45, 52, 120
Rat-Ia-C & Rat-S	1	0.03	77
Fugu-L & Pig-L	1	0.04*	89
Chicken-L & Salmon-L	1	0.049	108
Rainbow trout-L1 & Zebrafish-L1	3	0.03	45, 100, 114
Stickleback-L & Rat-Ia-C	1	0.048	125
			
**B. Convergent changes**			
			
Human-Ib-N & Rat-Ib-C	1	0.029	5
Cow-S & Frog-L	2	0.0071	12, 125
Pig-S & Frog-S1	2	0.002	15, 123
Fugu-L & Hagfish-S	2	0.007	21, 78
Frog-S2 & Rat-S	1	0.02*	100
Mouse-L & Killifish-L	1	0.02	38
Mouse-L & Rat-Ia-N	1	0.05	38
Rat-Ia-N & Killifish-L	1	0.035	38
Carp-L & Hagfish-S	2	0.007	41, 140
Mouse-Ia-N & Pig-S	1	0.016	43
Mouse-S & Mouse-Ib-C	1	0.014	52
Rat-Ib-N & Pig-S	1	0.02	54
Rainbow trout-L1 & Mouse-Ia-N	1	0.02	77
Fugu-L & Pig-L	1	0.04*	78
Pig-L & Human-Ia-C	1	0.02	96
Rat-S & Rainbow trout-L1	1	0.05	100
Mouse-L & Zebrafish-L1	1	0.048	101
Rat-S & Cow-S	1	0.015	101
Human-S & Salmon-L	1	0.008	108
Pig-S & Salmon-L	1	0.008	108
Human-S & Chicken-L	2	0.007	105, 108
Frog-S1 & Mouse-Ia-C	1	0.038	109
Mouse-L & Mouse-Ia-N	1	0.02	117
Human-Ia-C & Rat-S	1	0.03	121
Carp-L & Fugu-L	1	0.021	123
Zebrafish-L2-N & Medaka-L1	1	0.009	145

n = Number of sites where parallel or convergent evolution occurred.

P = Observed probability of parallel or convergent change

* = Observed probability of parallel and convergent change

Internodal branches are omitted.

Among the six parallel sites and ten convergent sites, which occurred with a significance level of 1%, sites 45, 78, and 123 may be potential adaptive sites. Site 45 has three independent parallel changes of S (Ser) to A (Ala) only in fish *PGRP-L *(three spine stickleback, rainbow trout and zebrafish). Serine is the only amino acid residue present in this site in *PGRP-L *except for these fishes. Moreover, exclusively non-polar character of this site indicates that it may have an important role in the function of these proteins especially because this residue is involved in substrate binding. Site 78 has suffered one parallel and one convergent substitution. The chemical profile of this site in mammal *PGRP-S *is exclusively hydrophobic. The variability which results from the change of I (Ile) to the polar T (Thr) in pig and cow may suggest an effect on the structure since this amino acid is located in the hydrophobic core. At the site 123 there is a convergent change of A (Ala) or Y (Tyr) to V (Val) in pig and frog *PGRP-S*, respectively located in the hydrophobic core. Both substitutions create a possible adaptive site in *PGRP-S *gene.

**Figure 5:**
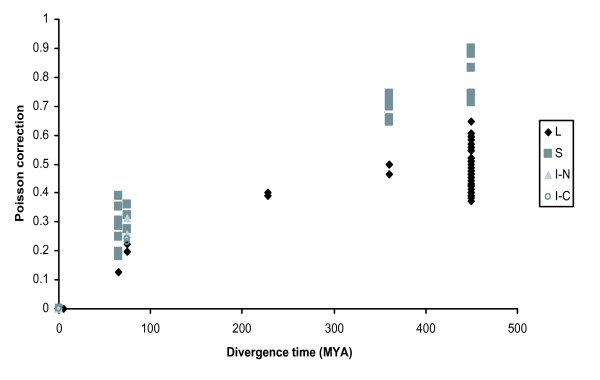
**Acceleration of evolutionary rate in the latest stages of vertebrate evolution**. Poisson distances of 19 taxa are plotted against species divergence time. The divergence times depicted in the abscissa correspond to: human-artiodactyls, human-mouse, human-chicken, human-frog; and human-fish with 65 MYA, 80 MYA, 228 MYA, 360 MYA and 450 MYA, respectively [38]. MYA: Million years ago.

In vertebrates, Tajima's relative test did not show rate heterogeneity in *PGRP-I *genes, but showed it in *PGRP-S *and *PGRP-L *genes [31] (Figure 5). We examined the genetic distances (poisson and gamma distances), their relationship between the divergence times, and found acceleration in the substitution rate in recent evolutionary stages.

**Figure 6:**
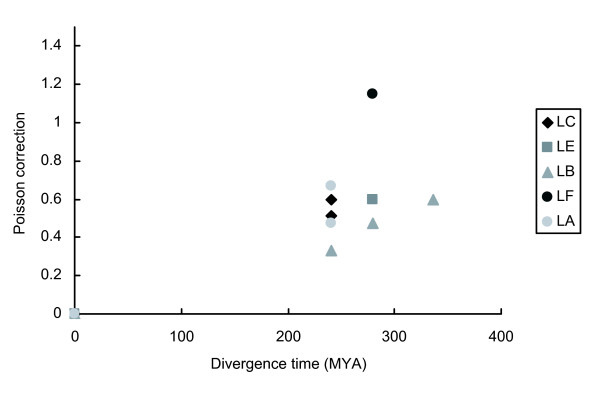
**Evolutionary rate in invertebrates**. Poisson distances of six taxa are plotted against species divergence time. The divergence times depicted in the abscissa correspond to: 241 MYA (Diptera - Hemiptera), 280 MYA (Diptera - Coleoptera), 336.1 MYA (Diptera - Lepidoptera). MYA: Million years ago.

We studied in invertebrates the genetic distances and the relationship between the divergence times and found that *PGRP-LC, PGRP-LE, PGRP-LB, PGRP-LF *and *PGRP-LA *have an evolutionary rate that is not constant (Figure 6).

## Discussion

### Evolutionary characteristics of PGRPs in vertebrates and invertebrates

When we compared PGRP evolution in vertebrates and invertebrates, we observed several differences, which are characteristic of each mode of evolution. First of all, despite the similar divergence time of 450 myr for the most recent common ancestor in each vertebrate and invertebrate (insect) species, the phylogenetic trees clearly show different patterns. In invertebrates each PGRP shows a relatively longer branch than those in vertebrates, suggesting a relatively ancient origin of each PGRP in invertebrates. In addition, in invertebrates the clustering pattern was rarely orthologous among *PGRP *genes, while in vertebrates orthologous relationship was clearly seen. This observation shows that higher rates of birth and death processes are seen in invertebrates than in vertebrates. Although the repertoire of PGRPs in each species may depend on some ecological and biological conditions, a less frequent birth and death process in vertebrates could reflect the presence of acquired immune system.

### Consequences of Natural Selection on PGRPs

In vertebrate PGRP proteins we observed changes as consequence of parallel and convergent amino acid substitutions, with significance greater than the random chance expectation. The changes may be either due to conservation of chemical property of amino acids at the site or due to modification of their properties. Convergent or parallel substitutions can provide evidence for the action of natural selection for keeping the function or structure [32].

PGRP proteins play an important role in innate immunity, which requires updated and immediate responses, because pathogens may change frequently. As a consequence, a high turnover rate is expected to happen. Actually, in invertebrates, the frequent turnover of PGRP repertoire was observed. On the other hand, several motifs indispensable for peptidoglycan recognition should be conserved through evolution of PGRPs in both vertebrates and invertebrates. In addition, we have observed that the amino acid residues that are located on the hydrophobic groove have high degree of conservation and do not show any parallel or convergent amino acid substitution.

### Evolution of the PGRP family in vertebrates and invertebrates and functional implications

This study provides for the first time a description of the origin and mode of evolution on vertebrate *PGRPs*, compared with invertebrates, namely insects, *PGRPs*.

*PGRPs *are proposed to be a family of genes that evolved by birth and death process with different rates in vertebrates and invertebrates. In the model of the birth and death process [33,34], some of the duplicated genes diverge functionally, but others become pseudogenes due to deleterious mutations or are deleted from the genome. The end result of this mode of evolution is a multi-gene family with a mixture of divergent groups of genes and highly homologous genes. We have observed that *PGRPs *have experienced several rounds of gene duplications and some duplicated genes have been deleted from the genome. This lineage specific birth and death process has been observed both in vertebrates and invertebrates.

The PGRP proteins are involved in innate immunity, which responds to protect the organisms from invading pathogens. Therefore, several motifs in PGRP domains are, of course, indispensable for pathogen recognition and have been conserved through the vertebrate and invertebrate evolution. However, since vertebrates possess acquired immunity, the significance of innate immunity might be more relaxed than in insects whose immune systems depend solely on innate immunity. This difference in the evolutionary patterns could be related to the plasticity of the receptors to detect a broad spectrum of microbial pathogens and it is clearly reflected in the birth and death process of the PGRP molecules in vertebrates and invertebrates.

## Conclusions

*PGRP *gene family reveals an example of genetic and functional variation of which roles in the immune systems are understood through an analysis of comparative genomics. Especially the analysis reveals that the mode of *PGRP *evolution was characterized by birth and death process. Vertebrates and invertebrates show striking differences in the evolutionary tempo and mode of *PGRP *genes. Broad repertoire of pathogen recognition proteins is advantageous in invertebrates, due to the absence of adaptive immunity, in contrast to the moderate repertoire in vertebrates. This reveals that the mode of evolution of a system strongly depends on other systems, which interact with the former both directly or indirectly.

## Methods

### Sequence Data

The sequences were retrieved from the genomic and EST NCBI database http://www.ncbi.nlm.nih.gov, the Ensembl database http://www.ensembl.org and the TIGR database http://www.tigr.org/tdb/tgi/ using TBLASTN and PSI-BLAST http://www.ncbi.nlm.nih.gov/BLAST/. Search was performed using each exon of human PGRP-S, PGRP-L, PGRP-Iα, and PGRP-Iβ as a probe. *Takifugu rubripes *PGRP-L full cDNA and genomic sequences were predicted using Genscan and confirmed by sequence analysis. *Takifugu rubripes *liver tissue was kindly provided by Dr. Shugo Watabe of the University of Tokyo, Japan. The *Eptatretus burgeri *PGRP cDNA sequence was kindly provided by Dr. Masanori Kasahara of The Graduate University for Advanced Studies (Sokendai), Hayama, Japan.

### GenBank accession numbers

The nomenclature used in this study and the accession numbers are listed on Additional Files 1 and 2.

### Rapid amplification of cDNA ends (RACE)

Based on the partial sequence information of chicken PGRP-L retrieved from databases, we reconstructed the missing 3' region of the transcript using the BD SMART™ RACE cDNA Amplification Kit (Clontech, USA) according to manufacturer's instructions.

### Data analyses

Sequences were aligned using the CLUSTALW version 1.83 computer program with its default parameter setting [35] and manually adjusted using the GeneDoc program version 2.6.002 [36]. Phylogenetical analyses were done using the neighbor-joining (NJ) and minimum evolution (ME) methods in MEGA version 4 [37]. The NJ and ME trees were based on the number of differences, and reliability was assessed by bootstrap values with 1000 replications. The reconciliation between species tree and gene tree along with the confirmation of the gene loss/duplication scenario were determined by using Notung 2.6 [25] and EvolMAP software [26]. To detect positive selection at single amino acid sites the Data Monkey software program was used with its default parameter setting http://www.datamonkey.org/. Poisson and gamma genetic distances were determined by using MEGA version 4 [37].

### Test of convergence

The ancestral sequences were determined using the program ANCESTOR [27], and the significance of the convergent and parallel sites was estimated using the program CONVERG2 [32].

## Abbreviations

The following abbreviations used in the manuscript are listed here in alphabetical order: ME: (minimum evolution); ML: (maximum-likelihood); NJ: (neighbor-joining); PGN: (peptidoglycan); PGRPs: (Peptidoglycan Recognition Proteins); RACE: (Rapid amplification of cDNA ends); SLAC: (Single Likelihood Ancestor Counting).

## Authors' contributions

AMM conceived, designed and performed the experiments. AMM, FT, YS and NT analyzed the data. AMM and YS wrote the paper. All authors read and approved the final manuscript.

## Supplementary Material

Additional file 1 **Table of vertebrate PGRP nomenclature**. Nomenclatures and resources of vertebrate PGRP sequences used in this study. Click here for file

Additional file 2 **Table of invertebrate PGRP nomenclature**. Nomenclatures and resources of invertebrate PGRP sequences used in this study. Click here for file

Additional file 3 **Alignment of vertebrate PGRPs**. Alignment of the C-terminal amino acid sequence of PGRPs from various vertebrate species. A dash represents the same amino acid as the above. Click here for file

Additional file 4 **Alignment of invertebrate PGRPs**. Alignment of the C-terminal amino acid sequence of PGRPs from various insects species. A dash represents the same amino acid as the above. Click here for file

Additional file 5 **Reconciled gene tree and species tree of vertebrate PGRPs**. NOTUNG analysis predicted 16 duplications and 42 losses. Two of the duplication events are domain duplications and three duplication events are possibly due to allelic divergence. D/L score = 66 [25]. Click here for file

Additional file 6 **Reconciled gene tree and species tree of invertebrate PGRPs**. NOTUNG analysis predicted 30 duplications and 53 losses. D/L score = 98 [25]. Click here for file

Additional file 7 **Average orthologs divergence tree of vertebrate PGRPs**. The EvolMAP analysis predicted 14 gains and 14 losses. In-paralogs, diverged in-paralogs and ambiguous gains constituted 36%, 57% and 7% of total gains, respectively. Gene gains (+) and gene losses (-) are depicted for each branch. Number of in-paralogs, diverged in-paralogs and ambiguous gains are indicated below or next to each gene gain [26]. Click here for file

Additional file 8 **Average orthologs divergence tree of invertebrate PGRPs**. The EvolMAP analysis predicted 26 gains and 8 losses. In-paralogs, and diverged in-paralogs gains constituted 27%, and 73% of total gains, respectively. Gene gains (+) and gene losses (-) are depicted for each branch. Number of in-paralogs, diverged in-paralogs and ambiguous gains are indicated below or next to each gene gain [26]. Click here for file

Additional file 9 **Alignment of PGRP ancestral sequences**. Alignment of ancestral sequences of *PGRP-L *and *PGRP-S*. A dash means the same amino acid as the above. A blue star indicates the amino acid position responsible for Zn^2+ ^ligand binding, whereas a red star indicates the amino acid position responsible for amidase activity. These sites are inferred from the sequences of T7 lysozyme of bacteriophage. Click here for file
