# Supplementary material for: Evolutionary origin of peptidoglycan recognition proteins in vertebrate innate immune system
Source: BMC Evol Biol. 2011 Mar 25;11:79. doi: 10.1186/1471-2148-11-79 (PMC3071341; doi:10.1186/1471-2148-11-79)
Supplement: Additional file 1 — Table of vertebrate PGRP nomenclature. Nomenclatures and resources of vertebrate PGRP sequences used in this study. [file 1471-2148-11-79-S1.PDF]

Notation	Common name	Species name	Accession number	Database
Bota-S	Cow	*Bos taurus*	AY083309	a
Cadr-S	Arabian camel	*Camelus dromedarius*	AJ131676	a
Cyca-L	Common carp	*Cyprinus carpio*	CA967718	a
Dare-F1	Zebrafish	*Danio renio*	ENSDARG00000023215	b
Dare-L1	Zebrafish	*Danio renio*	ENSDARG00000023725	b
Dare-L2	Zebrafish	*Danio renio*	ENSDARG00000015626	b
Hosa-L	Human	*Homo sapiens*	AF384856	a
Hosa-S	Human	*Homo sapiens*	NM_005091	a
Hosa-Ia	Human	*Homo sapiens*	NM_052891	a
Hosa-Ib	Human	*Homo sapiens*	AY035377	a
Mumu-L	Mouse	*Mus musculus*	NM_021319	a
Mumu-S	Mouse	*Mus musculus*	NM_009402	a
Mumu-Ia	Mouse	*Mus musculus*	AY518698	a
Mumu-Ib	Mouse	*Mus musculus*	NM_207263	a
Rano-S	Rat	*Rattus norvegicus*	AF154114	a
Rano-Ia	Rat	*Rattus norvegicus*	ENSRNOG00000012267	b
Rano-Ib	Rat	*Rattus norvegicus*	ENSRNOG00000021798	b
Epbu*	Inshore hagfish	*Eptatretus burgeri*	AB189841	a
Fuhe-L	Killifish	*Fundulus heteroclitus*	CN981276	a
Gaac-L	Three spined stickleback	*Gasterosteus aculeatus*	CD507320	a
Gaga-L*	Chicken	*Gallus gallus*	AY740510	a
Modo-S	Gray short tailed opossum	*Monodelphis domestica*	ENSMODT00000027381	b
Modo-L	Gray short tailed opossum	*Monodelphis domestica*	ENSMODT00000018587	b
Modo-I	Gray short tailed opossum	*Monodelphis domestica*	ENSMODT00000022114	b
Oidi	Tunicate	*Oikopleura dioica*	AAV73847	a
Onmy-L1	Rainbow trout	*Oncorhynchus mykiss*	CA374221	a
Onmy-L2	Rainbow trout	*Oncorhynchus mykiss*	BX912097	a
Onmy-L3	Rainbow trout	*Oncorhynchus mykiss*	BX869922	a
Onmy-L4	Rainbow trout	*Oncorhynchus mykiss*	BX316902	a
Orla-L1	Japanese medaka	*Oryzias latipes*	BJ746861	a
Orla-L2	Japanese medaka	*Oryzias latipes*	BJ716081	a
Patr-L	Chimpanzee	*Pan troglodytes*	ENSPTRG00000010624	b
Sasa-L	Atlantic salmon	*Salmo salar*	CA056926	a
Susc-L	Pig	*Sus scrofa*	NM_213738	a
Susc-S	Pig	*Sus scrofa*	NM_001001260	a
Taru-F1	Torafugu	*Takifugu rubripes*	SINFRUG00000131526	b
Taru-L	Torafugu	*Takifugu rubripes*	SINFRUG00000154623	b
Xela-S	African clawed frog	*Xenopus laevis*	CF523689	a
Xetr-S	Western clawed frog	*Xenopus tropicalis*	fgenesh_abini.C_scaffold_5400000001	b
Xetr-L	Western clawed frog	*Xenopus tropicalis*	fgenesh_abini.C_scaffold_403000031	b

*New sequences used in this study

a: NCBI database, b: Ensembl database
