# Supplementary material for: Evolutionary origin of peptidoglycan recognition proteins in vertebrate innate immune system
Source: BMC Evol Biol. 2011 Mar 25;11:79. doi: 10.1186/1471-2148-11-79 (PMC3071341; doi:10.1186/1471-2148-11-79)
Supplement: Additional file 2 — Table of invertebrate PGRP nomenclature. Nomenclatures and resources of invertebrate PGRP sequences used in this study. [file 1471-2148-11-79-S2.PDF]

Notation	Common name	Species name	Accession number	Database
Anga-PGRPLA	Mosquito	*Anopheles gambiae*	ENSANGT0000001044	a
Anga-PGRPLB	Mosquito	*Anopheles gambiae*	ENSANGT00000013948	a
Anga-PGRPLC3	Mosquito	*Anopheles gambiae*	ENSANGT00000029002	a
Anga-PGRPS2	Mosquito	*Anopheles gambiae*	ENSANGT00000012978	a
Anga-PGRPS3	Mosquito	*Anopheles gambiae*	ENSANGT00000012979	a
Anga-PGRP7	Mosquito	*Anopheles gambiae*	ENSANGT00000030333	a
Apme-PGRP1	Honey Bee	*Apis mellifera*	XP_395941	b
Apme-PGRP2	Honey Bee	*Apis mellifera*	XP_392452	b
Apme-PGRP3	Honey Bee	*Apis mellifera*	XP_001121036	b
Apme-PGRP4	Honey Bee	*Apis mellifera*	XP_001123180	b
Bomo-PGRP1	Domestic silkworm	*Bombyx mori*	NP_001036836	b
Bomo-PGRP2	Domestic silkworm	*Bombyx mori*	NP_001037560	b
Bomo-PGRP3	Domestic silkworm	*Bombyx mori*	NP_001036858	b
Bomo-PGRP4	Domestic silkworm	*Bombyx mori*	AADK01016620	b
Bomo-PGRP5	Domestic silkworm	*Bombyx mori*	AADK01003610	b
Bomo-PGRP6	Domestic silkworm	*Bombyx mori*	AADK01003610	b
Bomo-PGRP7	Domestic silkworm	*Bombyx mori*	AADK01011141	b
Bomo-PGRP9	Domestic silkworm	*Bombyx mori*	BAAB01095230, AADK01044291,	b
			BAAB01122160	b
Bomo-PGRP12	Domestic silkworm	*Bombyx mori*	AADK01012144	b
Drme-PGRPSA	Fruit fly	*Drosophila melanogaster*	NP_572727	b
Drme-PGRPSB1	Fruit fly	*Drosophila melanogaster*	CAD89138	b
Drme-PGRPSB2	Fruit fly	*Drosophila melanogaster*	CAD89150	b
Drme-PGRPSC1a	Fruit fly	*Drosophila melanogaster*	CAD89163	b
Drme-PGRPSC1b	Fruit fly	*Drosophila melanogaster*	CAD89174	b
Drme-PGRPSD	Fruit fly	*Drosophila melanogaster*	CAD89193	b
Drme-PGRPLA	Fruit fly	*Drosophila melanogaster*	NP_996026	b
Drme-PGRPLB	Fruit fly	*Drosophila melanogaster*	NP_731575	b
Drme-PGRPLC	Fruit fly	*Drosophila melanogaster*	NP_729468	b
Drme-PGRPLD	Fruit fly	*Drosophila melanogaster*	NP_001027111	b
Drme-PGRPLE	Fruit fly	*Drosophila melanogaster*	NP_573078	b
Drme-PGRPLF	Fruit fly	*Drosophila melanogaster*	NP_648299	b
Hodi-PGRP1	Korean black chafer	*Holotrichia diomphalia*	BAD08316	b
Hodi-PGRP2	Korean black chafer	*Holotrichia diomphalia*	BAD08317	b
Hodi-PGRP3	Korean black chafer	*Holotrichia diomphalia*	BAD08318	b
Trca-PGRP1	Red flour bettle	*Tribolium castaneum*	XP_969883	b
Trca-PGRP2	Red flour bettle	*Tribolium castaneum*	XP_972267	b
Trca-PGRP3	Red flour bettle	*Tribolium castaneum*	XP_972322	b
Trca-PGRP4	Red flour bettle	*Tribolium castaneum*	GLEAN_02790	c
Trca-PGRP5	Red flour bettle	*Tribolium castaneum*	XP_969556	b
Trca-PGRP6	Red flour bettle	*Tribolium castaneum*	XP_969402	b
Trca-PGRP7	Red flour bettle	*Tribolium castaneum*	XP_968926	b
Trca-PGRP8	Red flour bettle	*Tribolium castaneum*	XP_970847	b

a: Ensembl database, b:NCBI database, c:Tribolium castaneum Genome Project (Human Genome Sequencing Center at Baylor College of Medicine)
