# Supplementary material for: Evolutionary origin of peptidoglycan recognition proteins in vertebrate innate immune system
Source: BMC Evol Biol. 2011 Mar 25;11:79. doi: 10.1186/1471-2148-11-79 (PMC3071341; doi:10.1186/1471-2148-11-79)
Supplement: Additional file 3 — Alignment of vertebrate PGRPs. Alignment of the C-terminal amino acid sequence of PGRPs from various vertebrate species. A dash represents the same amino acid as the above. [file 1471-2148-11-79-S3.PDF]

Gaac-L	PRCQWRAKAHQGTPIPLSIPLQFLYVHHTYEPSSPCLTFPNCSRDMRAMQ
Onmy-L1	PRCQWGAAPYRGSPFPLALPLPFLYIHHTYEPDRPCHSFRQCSRSMRAMQ
Onmy-L3	PRCQWGAAPYRGTPTPLSLPLSFMYIHHTYQPGQPCLTFQQCSADMSSMQ
Onmy-L4	PRCQWGAAPYRGTPTPLSLPLSFMYIHHTYQPGQPCLTFQQCSADMRSMQ
Sasa-L	PRCQWGAAPYRGTPTPLSLPLSFMYIHHTYQPGQPCLTFQQCSADMRSMQ
Orla-L1	ARCTWGAAPYIGTPTMLSLPLTYLFIHHTASPSQPCLTFEQCSADMRSMQ
Orla-L2	ARCTWGAAPYIGTPTMLSLPLTYLFIHHTASPSQPCLTFEQCSADMRSMQ
Fuhe-L	PRCQWGAKANRDTPIPLSLPLQFLYVHHTYEPSSPCLSFSSCSRNMRSMQ
Xetr-S	SRSSWGGVPSKCQAK-LPRSVKYVIIHHTAG--ASCNSESACKAQARNIQ
Epbu	NREEWGARSPNETTT-LTPPQPLVIIHHTNW--NSCWTLEQCKSEVRKVQ
Hosa-S	PRNEWKALASECAQH-LSLPLRYVVVSHTAG--SSCNTPASCQQQARNVQ
Susc-S	SRREWGALASECSAN-LRRPVRYVVVSHTAG--STCDNPASCRRQVQNVQ
Cadr-S	PRREWRALASECRER-LTRPVRYVVVSHTAG--SHCDTPASCAQQAQNVQ
Bota-S	SRGKWGALASKCSQR-LRQPVRYVVVSHTAG--SVCNTPASCQRQAQNVQ
Rano-S	PRSEWKALPSECSKG-LKKPVRYVVISHTAG--SFCSSPDSCEQQARNVQ
Mumu-S	PRSEWRALPSECSSR-LGHPVRYVVISHTAG--SFCNSPDSCEQQARNVQ
Modo-S	PRSEWGALPSSCLKP-LNLPVEYVVVSHTAG--QPCNSASSCEQQMRNIQ
Xela-S	TKAQWGGRAATCRTA-MTTPVPYVIIHHTAG--AHCSSQTSCISQAKSIQ
Dare-F1	SRRGWDAVQPREMTQ-MESPAHTVIVHHTAL--RFCAHPRESVTELAHIQ
Furu-F1	SRAQWGAAPPKRREN-LTGPAQKVVIHHTAL--PKCSGLSGCRDRLLSIQ
Hosa-Ia-N	SRKEWGARPLACRAL-LTLPVAYIITDQLPG--MQCQQQSVCSQMLRGLQ
Rano-Ia-N	SRKVWGASSLTCRVP-LSLPVPYLIIEQVTR--MQCQDQTSCSQVLRVLH
Mumu-Ia-N	SRKEWGASSLTCRVP-LSLPVPYLIIEQVTR--MQCQDQITCSQVVRVLQ
Hosa-Ib-N	SRKAWGAEAVGCSIQ-LTTPVNVLVIHHVPG--LECHDQTVCSQRLRELQ
Rano-Ib-N	SRKGWGAEATGCSSK-LGRPVDVLVIHHVPG--LECHNQTVCSQKLRELQ
Mumu-Ib-N	SREEWGAEAIGCSSK-LSRPVDVLVIHHIPG--LECHNKTVCSQKLRELQ
Modo-I-N	SRSEWGKGPSGCNIQ-LRTPVPYLIIHHILG--QECHEKATCRQRVKGLQ
Hosa-Ia-C	KRSAWEARETHCPK--MNLPAKYVIIIHTAG--TSCTVSTDCQTVVRNIQ
Rano-Ia-C	PRTAWEARETHCSQ--MNLPAKFVIIIHTAG--ESCNESADCLIRVRDTQ
Mumu-Ia-C	PRSAWEARETHCPQ--MNLPAKFVIIIHTAG--KSCNESADCLVRVRGTQ
Hosa-Ib-C	PRSVWGARETHCPR--MTLPAKYGIIIHTAG--RTCNISDECRLLVRDIQ
Rano-Ib-C	PRSAWGARESHCFK--MTLPAKYAIILHTAG--RTCSQPDECRLLIQDLQ
Mumu-Ib-C	PRSVWGARDSHCSR--MTLPAKYAIILHTAG--RTCSQPDECRLLVRDLQ
Modo-I-C	PRSSWGAQDTDCSK--LPGPAKYVVIIHTGG--RNCNETEECQIALRYIQ
Dare-L2-N	TRSQWGAASYIGSPSYLSLPVRYLFIHHTYQPSKPCTTFEQCAAEMRSMQ
Dare-L2-C	TRSQWGAASYIGSPSYLSLPVRYLFIHHTYQPSKPCTTFEQCAAEMRSMQ
Susc-L	PRCRWGAAPYRGSPKPLKLPLGFLYIHHTYVPARPCTDFALCAANMRSMQ
Patr-L	PRCRWGAAPYRGRPKLLQLPLGFLYVHHTYVPAPPCTDFTRCAANMRSMQ
Hosa-L	PRCRWGAAPYRGRPKLLQLPLGFLYVHHTYVPAPPCTDFTRCAANMRSMQ
Mumu-L	PRCRWGAAPYRGHPTPLRLPLGFLYVHHTYVPAPPCTTFQSCAADMRSMQ
Modo-L	PRCRWGAAPYRGSPTMLNLPLGFLYVHHTYEPHQPCTSFSQCAANMKSMQ
Xetr-L	PRCMWGAKRYKGKPIFLGLPLSRVFIHHTYEPSQPCTSFSQCAANMRSMQ
Gaga-L	PRCMWGARPYRGTPRPLSPPLGSIYIHHTFVPSAPCRSFTACARDMRSMQ
Dare-L1	PRCIWGAAPPQVPLELLSPPMSFLYIHHTAIPSKPCLNLQTCSQNMRAMQ
Taru-L	PRCQWGAEAHRGTPMPLSLPLPFLYVHHTYEPSSPCLSFPNCSHDMRSMQ
Cyca-L	SRSQWGAAAFIGSPSYLSLPMPYLFIHHTYQPSKPCTTFDQCDSDMRSMQ
Onmy-L2	PRCQWGAAPYRGTPTPLSLPLSFMYIHHTYQPGQPCLTFQQCSADMRSMQ
Oidi	PRAHWEARLPLGIDNYFHYDGIGVIGHHTHW-DR-CFDIVDCIKEVKKVQ

Gaac-L	RFHQEDRGWEDIG-----------YSFVVGSDGYVYEGRGWKHLGRH-TR
Onmy-L1	RFHQEDRGWADIG-----------YSFVVGSDGYIYEGRGWYHLGTH-TR
Onmy-L3	RFHQDDRGWDDIG-----------YSFVAGSDGYLYEGRGWHWQGAH-TK
Onmy-L4	RFHQDDRGWDDIG-----------YSFVAGSDGYLYEGRGWNWQGAH-TK
Sasa-L	RFHQDDRGWDDIG-----------YSFVAGSDGYLYEGRGWHWQGAH-TK

```
Orla-L1        RFHQQTNGWDDIG-----------YSFVAGSDGNIYEGRGWKWQGAH-TG
Orla-L2        RFHQQTNGWDDIG-----------YSFVAGSDGNIYEGRGWKWQGAH-AG
Fuhe-L         RFHQDDRGWNDIG-----------YSFVVGSDGYIYEGTGWNHVGRH-TR
Xetr-S         NFHMKSNGWCDTG-----------YNFLIGEDGQVYEGRGWETVGAH-AK
Epbu           DFHMDVRNWWDIG-----------YNFLVGEDGRAYEGRGWTTEGAH-AE
Hosa-S         HYHMKTLGWCDVG-----------YNFLIGEDGLVYEGRGWNFTGAHSGH
Susc-S         HYHVRTLHWCDVG-----------YNFLIGEDGLVYEGRGWNTVGAHSGP
Cadr-S         SYHVRNLGWCDVG-----------YNFLIGEDGLVYEGRGWNIKGAHAGP
Bota-S         YYHVRERGWCDVG-----------YNFLIGEDGLVYEGRGWNTLGAHSGP
Rano-S         LYQMKQLGWCDVA-----------YNFLIGEDGHVYEGRGWTIKGDHTGP
Mumu-S         HYHKNELGWCDVA-----------YNFLIGEDGHVYEGRGWNIKGDHTGP
Modo-S         FYHINTLGWCDIA-----------YNFLIGEDGLIYEGRGWSTLGAHTGP
Xela-S         NYHMNSNAWCDVG-----------YSFLVGEDGNVYEGRGWNSVGAH-AP
Dare-F1        RMHMQERGFDDIG-----------YNFLISGDGTVYEGRGWGIVGAH-AK
Furu-F1        RSHMNDRNFDDIG-----------YNFLVGFDGTVFEGRGWGVVGAH-AK
Hosa-Ia-N      SHSVYTIGWCDVA-----------YNFLVGDDGRVYEGVGWNIQGLH-TQ
Rano-Ia-N      SHYVHNKGWCDVA-----------FNFLVGNDGKVYEGVGWHVQGLH-TQ
Mumu-Ia-N      SQYVHNKGWCDIA-----------FNFLVGDDGKVYEGVGWYVQGLH-TQ
Hosa-Ib-N      AHHVHNNSGCDVA-----------YNFLVGDDGRVYEGVGWNIQGVH-TQ
Rano-Ib-N      AYHIRN-HWCDVA-----------YNFLVGDDGKVYEGVGWNVQGSH-DQ
Mumu-Ib-N      AYHIHN-SWCDVA-----------YNFLVGDDGRVYEGVGWNVQGSH-DQ
Modo-I-N       ELHIKINGWCDVA-----------YNFLIGEDGNVYEGLGWTLEGTH-TM
Hosa-Ia-C      SFHMDTRNFCDIG-----------YHFLVGQDGGVYEGVGWHIQGSH-TY
Rano-Ia-C      SFHMDKQDFCDIA-----------YHFLVGQDGVVYEGVGWTIEGSH-TY
Mumu-Ia-C      SFHIDNQDFCDIA-----------YHFLVGQDGEVYEGVGWNIEGSH-TY
Hosa-Ib-C      SFYIDRLKSCDIG-----------YNFLVGQDGAIYEGVGWNVQGSS-TP
Rano-Ib-C      SFFMDRLNACDIG-----------YNFLVGQDGGVYEGVGWNNQGSK-TD
Mumu-Ib-C      SFFMNRLNACDIG-----------YNFLVGQDGGVYEGVGWNNQGSK-TD
Modo-I-C       SYHIEKMKFCDIA-----------YNFLVGEDGKAYEGVGWDTEGAH-TY
Dare-L2-N      RYHQQSNGWSDIG-----------YSFVAGSDGNLYEGRGWNWVGAH-TY
Dare-L2-C      RYHQQSNGWSDIG-----------YSFVAGSDGNLYEGRGWNWVGAH-TY
Susc-L         RFHLDTQGWDDIG-----------YSFVVGSDGYVYEGRGWHWVGAH-TR
Patr-L         RYHQDTQGWGDIG-----------YSFVVGSDGYVYEGRGWHWVGAH-TL
Hosa-L         RYHQDTQGWGDIG-----------YSFVVGSDGYVYEGRGWHWVGAH-TL
Mumu-L         RFHQDVRKWDDIG-----------YSFVVGSDGYLYQGRGWHWVGAH-TR
Modo-L         RYHQDTNGWDDIG-----------YSFVVGTDGYVYEGRGWHWVGAH-TL
Xetr-L         RFHQQDRGWDDIGYRTCNRSSPLSYSFVVGSNGYLYEGRGWNRAGAH-TR
Gaga-L         RFHQDTRGWDDIG-----------YSFVVGSDGYLYQGRGWRWVGAH-TR
Dare-L1        RFHQKDRGWYDIG-----------YSFVVGSDGYIYEGRGWMSQGAH-TK
Taru-L         RFHQDDRGWNDIG-----------YSFVVGSDGYIYEGRGWNTLGSH-TR
Cyca-L         RYHQQTNGWSDIG-----------YSFVAGSDGNLYEGRGWNWVGAH-TY
Onmy-L2        RFHQDDRGWDDIG-----------YSFVAGSDGYLYEGRGWHWQGAH-TK
Oidi           DYHMDGNGWWDVG-----------YNFLIGEDGRIYEGRG-----AH-CS

Gaac-L         GHNAIGYGVSIIGNYTATLPSRHAADLLRHRLVRCAVDGGWLAAN-FTIQ
Onmy-L1        GQNPYGYGVAFIGNYSSSLPSLHALDLVRQHLAKCAVDGGRLQAN-FTLH
Onmy-L3        GYNSKGYGVSFIGDYTSSLPSEQTMELVRDRLASCAVGGGRLVGN-FTLY
Onmy-L4        GYNSKGYGVSFIGDYTSSLPSEQTMELVRDRLASCAVGGGRLVGN-FTLY
Sasa-L         GYNSKGYGVSFIGDYTSSLPSQQTIQLVRDRLASCAVGGGRLVGN-FTLY
Orla-L1        GYNSKGYGVSFIGDYTSTLPSQHAMALVRDQLASCAVAGGQLVSS-YILK
Orla-L2        GYNSKGYGVSFIGDYTSTLPSQHAMALVRDQLVSCAVAGGQLVSS-YILK
Fuhe-L         GHNSIGYGVSIIGNYTVTLPSRHAMDLLRHKLALCAVNGGVLTAN-FTIQ
Xetr-S         NYNFNSIGISFMGTFTNRAPNTAAQKAAKD-LISCGVAKKVINSD-YTLK
Epbu           GYNNCSIGISVMGTFEDAAPNITALHTLQK-IITFGVTEGFIDLN-YTLY
Hosa-S         LWNPMSIGISFMGNYMDRVPTPQAIRAAQG-LLACGVAQGALRSN-YVLK
Susc-S         TWNPLSLGISFMGNYMNRVPPARAIRAAQS-LLACGVALGVLRPN-YEVK
Cadr-S         TWNPISIGISFMGNYMNRVPPPRALRAAQN-LLACGVALGALRSN-YEVK
```

```
Bota-S          TWNPIAIGISFMGNYMHRVPPASALRAAQS-LLACGAARGYLTPN-YEVK
Rano-S          IWNPMSIGITFMGDYSHRVPAKRALRAALN-LLKCGVSEGFLRSN-YEVK
Mumu-S          IWNPMSIGITFMGNFMDRVPAKRALRAALN-LLECGVSRGFLRSN-YEVK
Modo-S          AWNPISLGISFIGNFMERAPSPRALRAAQS-LISCGLQHQAIARQ-YMIK
Xela-S          NYNSNSIGISVMGTYTNINPNTAAQNAVKN-LISCGVTKGYIKST-YILK
Dare-F1         EHNFYSVGIAFMGNFNADLPSSASLSALLR-LLHIGVLHGHVRPN-FVLL
Furu-F1         GFNNESLGIAFMGNFNNDTPSSEAVLSVRQ-LLHSGVSQGFLCPD-FALM
Hosa-Ia-N       GYNNISLGIAFFGNKIGSSPSPAALSAAEG-LISYAIQKGHLSPR-YIQP
Rano-Ia-N       GYNNVSLGIAFFGSKIGSSPSPAALSATED-LIFFAILNGYLSPK-YIQP
Mumu-Ia-N       GYNNVSLGIAFFGSKIG-SPSPAALSATED-LIFSAIQNGHLSPK-YTQP
Hosa-Ib-N       GYNNISLGFAFFGTKKGHSPSPAALSAMEN-LITYAVQKGHLSSS-YVQP
Rano-Ib-N       GYNNISLGVAFFGTQEGHSPSPVALLAMEA-LISHAVKKGHLSSK-YIQP
Mumu-Ib-N       GYKNISLGVAFFGTQEGHSPSPVALSAMKG-LISYAVKKGHLSSK-YIQP
Modo-I-N        GYNRKSLGFAFVGSAAGSSPSAAALTAAEN-LISFAVYNGYLSPK-YI--
Hosa-Ia-C       GFNDIALGIAFIGYFVEKPPNAAALEAAQD-LIQCAVVEGYLTPN-YLLM
Rano-Ia-C       GYNDIALGIAFMGNFVEKPPNEASLEAAQS-LIQCAVAMGYLASN-YLLM
Mumu-Ia-C       GYNDIALGIAFMGNFVEKPPNEASLKAAQS-LIQCAVAKGYLTSN-YLLM
Hosa-Ib-C       GYDDIALGITFMGTFTGIPPNAAALEAAQD-LIQCAMVKGYLTPN-YLLV
Rano-Ib-C       GYNDIALSIAFMGIFTGSSPNAAALQAAQD-LIQCAVVKGYLTPN-YLLM
Mumu-Ib-C       SYNDISLSITFMGTFTGSPPNAAALEAAQD-LIRCTVVKGYLTPN-YLLM
Modo-I-C        GYNDIGLGIAFMGLFTDNPPNDAALKAAQD-LIQCSVDKGYLDPD-YLLV
Dare-L2-N       GYNSIGYGVCFIGDYTSTLPASSAMNMVRYDFTYCATNGGRLSKS-YSLY
Dare-L2-C       GYNSIGYGVCFIGDYTSTLPASSAMNMVRYDFTYCATNGGRLSKS-YSLY
Susc-L          DHNSRGFGVALIGNYTAELPSEAALRAVRDELPHCAVRAGLLQPD-YALL
Patr-L          GHNSRGFGVAIVGNYTAALPTEAALRTVRDTLPSCAVRAGLLRPD-YALL
Hosa-L          GHNSRGFGVAIVGNYTAALPTEAALRTVRDTLPSCAVRAGLLRPD-YALL
Mumu-L          GYNSRGFGVAFVGNYTGSLPNEAALNTVRDALPS-AIRAGLLRPD-YKLL
Modo-L          GHNFLGFGVSFIGNYTATLPTAYALRIVRDTLPRCGVRAGHLRPD-YKIH
Xetr-L          GYNSVGYGVSFIGDYTSIVPKDSILALVKDRFLRCAVRLGYITPN-YIIQ
Gaga-L          GHNTKGYGVGYVGNFSASLPDPEAIALVRDGLIPCAVRAGWLHQN-YTLH
Dare-L1         GRNNVGYGVAFIGDYSGRLPSTHDMELVRHHLVKCGVNNGFLQED-FTIL
Taru-L          GHNSLGYGVSIIGNYTATLPSRHAMDLLRHRLVRCAISRGGLTPN-FTIH
Cyca-L          EYNSKGYGVSFIGDYTSTLPIKSAMDMVRYDFTSCAVNSGGLSSS-YSLY
Onmy-L2         GYNSKGYGVSFIGDYTSSLPSEQTMELVRDRLASCAVGGGRLVGN-FTLY
Oidi            GWNTQTLGFTIMGSFISDLPNSRALNAAKQ-LMREMEKRGFIDERCWSFF

Gaac-L          GHRQAVNYTSCPGDALFSEIRGWEHFGE----------------------
Onmy-L1         GHRQLVD-TSCPGDALYSEIKGWEHFGETSSSKKDQ--------------
Onmy-L3         GHRQLVK-TSCPGDAFYSEITGWEHFGEVQN-------------------
Onmy-L4         GHRQLVK-TSCPGDAFYSEITGWEHFGEVQN-------------------
Sasa-L          GHRQLVK-TSCPGDAFYSEITGWEHFGEVQN-------------------
Orla-L1         GHRQMVS-TECPGNIFYKEITTWEHYQP----------------------
Orla-L2         GHRQMVS-TECPGNIFYKEITTWEHYQP----------------------
Fuhe-L          GHRQVVNYTSCPGDA-----------------------------------
Xetr-S          GHRDVSA-TECPGTNLYNLIKNWPNFKA----------------------
Epbu            GHRQTGP-TDCPGQTFYNILQNGSHW------------------------
Hosa-S          GHRDVQR-TLSPGNQLYHLIQNWPHY------------------------
Susc-S          GHRDVQP-TLSPGDQLYEIIQKWPHYRA----------------------
Cadr-S          GHRDVQP-TLSPGDRLYEIIQTWSHYRA----------------------
Bota-S          GHRDVQQ-TLSPGDELYKIIQQWPHYRRV---------------------
Rano-S          GHRDVQS-TLSPGDQLYEIIQSWDHYRE----------------------
Mumu-S          GHRDVQS-TLSPGDQLYQVIQSWEHY------------------------
Modo-S          GHRDVQN-TASPGDKLYAKLRTWPHY------------------------
Xela-S          GHRNVGS-TECPGNTFYNTVKTWPRF------------------------
Dare-F1         GHKDVAK-TACPGENLYSVLPKLRDR------------------------
Furu-F1         GHRDLSA-TECPGANLYAALPKLKLR------------------------
Hosa-Ia-N       LLLKEET-CLDPQHPVMPR-------------------------------
```

```
Rano-Ia-N      FLLKEET-CLVPQHSEIPK-------------------------------
Mumu-Ia-N      FLLKEET-CLVPQHSEIPK-------------------------------
Hosa-Ib-N      LLVKGEN-CLAPRQKTSLK-------------------------------
Rano-Ib-N      LLVKSED-CLVPPQKGKQK-------------------------------
Mumu-Ib-N      LLAKSED-CLVPPQKGKQK-------------------------------
Modo-I-N       --------------------------------------------------
Hosa-Ia-C      GHSDVVN-ILSPGQALYNIISTWPHF------------------------
Rano-Ia-C      GHSDVSN-ILSPGQALYNIIKTWPHFKH----------------------
Mumu-Ia-C      GHSDVSN-ILSPGQALYNIIKTWPHFKH----------------------
Hosa-Ib-C      GHSDVAR-TLSPGQALYNIISTWPHF------------------------
Rano-Ib-C      GHSDVSN-TLSPGQALYNIIKTWPHFKH----------------------
Mumu-Ib-C      GHSDVSN-TLSPGQALYNIIKTWPHF------------------------
Modo-I-C       GHSDVVN-TLSPGQALYDQIKTWPHF------------------------
Dare-L2-N      GHRQAAA-TELCPNI-----------------------------------
Dare-L2-C      GHRQAAA-TECPGNTLYRQIQTWERYQ-----------------------
Susc-L         GHRQLVR-TDCPGDALFNMLRTWPRFNMNVKPRTARRASGRSKRRLPLMI
Patr-L         GHRQLVR-TDCPGDALFDLLRTWPHFTATVKPRPARSVSERSRREPPPRT
Hosa-L         GHRQLVR-TDCPGDALFDLLRTWPHFTATVKPRPARSVSKRSRREPPPRT
Mumu-L         GHRQLVL-THCPGNALFNLLRTWPHFTEVEN-------------------
Modo-L         GHRQLVH-TDCPGDALYRHIRTWPHF------------------------
Xetr-L         GHRQVVS-TSCPGDALYKEIQSWDHFKES---------------------
Gaga-L         GHRQMVN-TSCPGDALFQEIQTWHGFK-----------------------
Dare-L1        GHRQVVVTTSCPGNALYSEITTWMHY------------------------
Taru-L         GHRQVVN-TSCPGDAFFSEIQSWEHF------------------------
Cyca-L         GHRQATS-TDCPGNSFYREIQTWEHYQ-----------------------
Onmy-L2        GHRQLVK-TSCPGDAFYSEITGWEHFGEVQN-------------------
Oidi           GHRDKGN-TTCPGDRLFEEFKEWKNF------------------------

Gaac-L         -------
Onmy-L1        -------
Onmy-L3        -------
Onmy-L4        -------
Sasa-L         -------
Orla-L1        -------
Orla-L2        -------
Fuhe-L         -------
Xetr-S         -------
Epbu           -------
Hosa-S         -------
Susc-S         -------
Cadr-S         -------
Bota-S         -------
Rano-S         -------
Mumu-S         -------
Modo-S         -------
Xela-S         -------
Dare-F1        -------
Furu-F1        -------
Hosa-Ia-N      -------
Rano-Ia-N      -------
Mumu-Ia-N      -------
Hosa-Ib-N      -------
Rano-Ib-N      -------
Mumu-Ib-N      -------
Modo-I-N       -------
Hosa-Ia-C      -------
Rano-Ia-C      -------
```

```
Mumu-Ia-C      -------
Hosa-Ib-C      -------
Rano-Ib-C      -------
Mumu-Ib-C      -------
Modo-I-C       -------
Dare-L2-N      -------
Dare-L2-C      -------
Susc-L         PLATDLQ
Patr-L         LPATDLQ
Hosa-L         LPATDLQ
Mumu-L         -------
Modo-L         -------
Xetr-L         -------
Gaga-L         -------
Dare-L1        -------
Taru-L         -------
Cyca-L         -------
Onmy-L2        -------
Oidi           -------
```
