# Supplementary material for: Evolutionary origin of peptidoglycan recognition proteins in vertebrate innate immune system
Source: BMC Evol Biol. 2011 Mar 25;11:79. doi: 10.1186/1471-2148-11-79 (PMC3071341; doi:10.1186/1471-2148-11-79)
Supplement: Additional file 4 — Alignment of invertebrate PGRPs. Alignment of the C-terminal amino acid sequence of PGRPs from various insects species. A dash represents the same amino acid as the above. [file 1471-2148-11-79-S4.PDF]

```
Bomo_PGRP6_partial          ---------------------PIRLVIV-QHT--VSPECD-TFQTCASQM
Bomo_PGRP9_partial          SREEWGARPPTMTSPLK--VSPVPIVVI-HHS-YIPKICL-VRADCERDL
Bomo_PGRP5_partial          -RGDWQAMRPYTMDFL---ELPVSFVII-GHT--VTQYCN-QKYDCIKKI
Bomo_PGRP7_partial          RRSDWQAMSPYSVDFL---DLPLSFVIV-GHS--ATNYCT-EKYECIKEM
Trca_PGRP1_partial          SRTRWGARTALEVDYA---LIPVENVVV-HHT--VTNTCS-TEEECAAIL
Trca_PGRP4_partial          NREQWGAKPPLNQTRL---QHPVDLVII-GHT--VTDFCW-NFEQCSHIV
Bomo_PGRP12_partial         TRAEWQAKTPVQTTPLK---TPVPYVVI-HHT-YIPGACH-TREQCSSAM
Trca_PGRP5_partial          PREGWHARPPTATEPM--AN-PVPFVIT-HHS-YIPPACH-TPEACVQSM
Trca_PGRP6_partial          SRSEWGARAPKSSQPL--AQKPAPFVVV-HHS--DGSNCL-SLQACKSRV
Trca_PGRP7_partial          ARRTWLAQPPLDPDDVKFFKKPPKFVII-CHS--ASEEAY-TQTDNNLLV
Trca_PGRP8_partial          VREQWQAHVPSSTMP-KLELPVRRVLFL-PAN---TTSCG-SKSHCAKVL
Apme_PGRP1                  SRSEWGARKPTTTIR-ALAQNPPPFVII-HHS--ATDSCI-TQAICNARV
Apme_PGRP3_partial          SRKEWQARPPVARELM-DDKPKP-YVVV-HHGG-IIQYCF-DVKTCSAIV
Apme_PGRP4_partial          KRNEWTNVQAKNINYL---IIPIPYVII-HHT--VSLECN-SKDTCISNI
Hodi_PGRP3                  SKNRWGGQQARKVEPT---TKPLKYVII-NHT--SGPSCV-DEIDCSRML
Hodi_PGRP2                  SKNRWGGQQASQVQYT---VKPLKYVII-HHT--STPTCT-NEDDCSRRL
Hodi_PGRP1                  SKRDWGGNAALRVGYT---SKPLERVVI-HHT--VTPECA-NEARCSSRM
Anga_PGRPS3                 TRAQWGARAANTAQLP---IRPAPWVVM-HHT--AGAACT-TDAACAQQM
Anga_PGRPS2                 TRAQWGARAASTSQLP---IRPAPWVVM-HHT--AGASCT-TDAACAQQM
Drme_PGRP-SA-RA             LKRQWGGKPSLGLHYQ---VRPIRYVVI-HHT--VTGECS-GLLKCAEIL
Drme_PGRP-SC2-RA            SKSEWGGRSATSKTSL---ANYLSYAVI-HHT--AGNYCS-TKAACITQL
Drme_PGRP-SC1a-RA           SKAEWGGRGAKWTVGL---GNYLSYAII-HHT--AGSYCE-TRAQCNAVL
Drme_PGRP-LF-RA_221C        TRPYWLAQPPIVPLTP--LKLPIESVRF-VAT--NTPSCF-TQAECTFRV
Drme_PGRP-SD-RA             TRAEWNAKPPNGAIDS--METPLPRAVI-AHT--AGGACA-DDVTCSQHM
Drme_PGRP-SC1b-RA           SKAEWGGRGAKWTVGL---GNYLSYAII-HHT--AGSYCE-TRAQCNAVL
Drme_PGRP-SB1-RA            PRSSWGAVSARSPSRI---SGAVDYVII-HHS-DNPNGCS-TSEQCKRMI
Drme_PGRP-SB2-RA            PRSSWCPVPISPR-MP-RLMVPVRLIII-HHT--VTAPCF-NPHQCQLVL
Anga_PGRPLB                 TRDFWSALP-PKR-IE-HFAGPIPYVII-HHS-YRPAACY-NGLQCIAAM
Drme_PGRP-LB-RC             SRSDWGARL-PKS-VE-HFQGPAPYVII-HHS-YMPAVCY-STPDCMKSM
Drme_PGRP-LF-RA_N220        DRSEWLGEPPSGK-YP-HLKLPVSNIII-HHT--ATEGCE-QEDVCIYRM
Trca_PGRP3_partial          EKKIWGGRATLNF-SK-PLPHPTHFVIV-SHT--VTPTCS-DFPACSQRV
Drme_PGRP-LD-i1             GHGIWSDMELQGRGTL-FDPIGVGTVIF-THTG-SNE-CH-D--DCPDVL
Apme_PGRP2                  ERKEWGAQPPTTQ-LI-KMKLPVPYVII-SHT--ATQFCS-TQSECTFYV
Bomo_PGRP4_partial          SRSDWLAQPVESKYLA-KLSHPVPWVII-SHT--ATESCS-NQSQCVLRV
Trca_PGRP2_partial          SRLEWLAQPPVQP-AN-PLAVPVPYVII-LHT--ATENCS-SQAQCIFHV
Anga_PGRPLC3                TRTEWLAQPPREE-LT-DLKLPVNNVII-AHT--ATEGCT-TQTKCMYQV
Drme_PGRP-LC-RA             KVAEWGGRPAKRM-LD-AQQLPINRVVI-SHT--AAEGCE-SREVCSARV
Drme_PGRP-LE-RA             PRSSWLAQKPMDE-PL-PLQLPVKYVVI-LHT--ATESSE-KRAINVRLI
Drme_PGRP-LA-RE             DREQWGASKNSHGLTI-PLKRPIPYVLI-THIGVQSLPCD-NIYKCSIKM
Anga_PGRPcan7_partial       DRHNWGAQQGVHG-PY-KLPHPIPYVLI-THIGVHSEICS-DVHVCSIKM
Anga_PGRPLA_partial         ERRNWGSQLDAHA-SI-QLEHPVQYVIV-THIGLRSKNCT-GMHECANRM
Bomo_PGRP1                  SKKQWDG-LIPVH-VS-YLARPVSLVIV-QHT-VTP-FCR-TDAGCEELV
Bomo_PGRP2                  PITEWSG-TESRR-KQ-PLKSPIDLVVI-QHT-VSN-DCF-TDEECLLSV
Bomo_PGRP3                  SRDCWGAVPSKDT-RP-LNK-PVPYVII-HHT-AIPTVCN-TTTQCMRDM

Bomo_PGRP6_partial          RILQSNVLNN--LED-DISYNFLIGNDGRVYEGRGWGLVGAHTYHY-NRC
Bomo_PGRP9_partial          RNMQRVHQV--TNGWEDIGYSFAVGGEGTVFEGRGWSSIGAHAFGV-NTR
Bomo_PGRP5_partial          INVQKSHLD--AR-FEDIGPNFLISGNGIVFEGRGANVLSTMLKGW-NRR
Bomo_PGRP7_partial          LDVQKSHLH--RG-WQDIGPNFLVSGNGIVFEGRGANVFGAMAIAW-NRR
Trca_PGRP1_partial          RNIQNFHME--NLDFHDIGYSFLVGGDGQIYEGAGWHKVGAHTRGY-NSR
Trca_PGRP4_partial          AAIQNYHLE---LKMSDIAYNFLIGGDGNIYEGRGWDVAN----SQ-RNH
Bomo_PGRP12_partial         RGMQNYSMI--DNNWWDIGYSFAVGGDG-VYEGRGWTDRGAHSLHF-NNI
Trca_PGRP5_partial          QTMQDMHQL--QNGWNDIGYSFGVGGDGNAYEGRGWSKVGAHAPKY-NNI
Trca_PGRP6_partial          KGIQNYHID--HNGWQDIGYNFQIGGDGNVYEGRGWGIWGAHVPRY-NSK
```

```
Trca_PGRP7_partial          RLIQQFHVE--SRKWNDISYNFLVGAEGSVYEGRGWKTVGAHTQGY-NSV
Trca_PGRP8_partial          QELQLQHML--QWKEPDISYNFIMTADGRIFEGRGWDFETSVQNCT-VND
Apme_PGRP1                  RSFQNYHID--EKGWGDIGYQFLVGEDGNIYEGRGWDKHGAHSISY-NSK
Apme_PGRP3_partial          REYQNMHLD--ERGWYDIGYSFVIGEDGNAYEGRGWDYVGAHAPGY-NTQ
Apme_PGRP4_partial          ENIRSYHMD--TLNWHDIGYRFLIGGDGNIYEGCGWNHEGAHTYGY-NKK
Hodi_PGRP3                  VYIQNRHMN--HLNYNDIGCNFIIGGDGQIYEGAGWQAAASHTPGW-NKK
Hodi_PGRP2                  VNIQDYHMN--RLDFDDIGYNFMIGGDGQIYEGAGWHKEGAHARGW-NSK
Hodi_PGRP1                  VSMQNYHMD--ELGYDDISYNFVIGGDGRVYEGVGWHKKGSHSPGW-DSQ
Anga_PGRPS3                 RNIQSFHMD--GNGWADIGYNFLVGENGAAYEGRGWGRQGAHAPGY-NDR
Anga_PGRPS2                 RNIQSFHMD--GNGWADIGYNFLVGENGAAYEGRGWGRQGAHAPGY-NDR
Drme_PGRP-SA-RA             QNMQAYHQN--ELDFNDISYNFLIGNDGIVYEGTGWGLRGAHTYGY-NAI
Drme_PGRP-SC2-RA            QNIQAYHMD--SLGWADIGYNFLIGGDGNVYEGRGWNVMGAHATNW-NSK
Drme_PGRP-SC1a-RA           QSVQNYHMD--SLGWPDIGYNFLIGGDGNVYEGRGWNNMGAHAAEW-NPY
Drme_PGRP-LF-RA_221C        RLLQNWHIE--SNGYKDINYNFVAAGDENIYEARGWD-HSCEPPK--DAD
Drme_PGRP-SD-RA             QNLQNFQMS--KQKFSDIGYHYLIGGNGKVYEGRSPSQRGAFAGPN-NDG
Drme_PGRP-SC1b-RA           QSVQNYHMD--SLGWPDIGYNFLIGGDGNVYEGRGWNNMGAHAAEW-NPY
Drme_PGRP-SB1-RA            KNIQSDHKG--RRNFSDIGYNFIVAGDGKVYEGRGFGLQGSHSPNY-NRK
Drme_PGRP-SB2-RA            RQIRADHM---RRKFRDIGYNFLIGGDGRIYEGLGFGIRGEHAPRY-NSQ
Anga_PGRPLB                 QSMQKMHQD--ERQWNDIGYSFAVGGDGHVYQGRGFNVIGAHAPRY-NNR
Drme_PGRP-LB-RC             RDMQDFHQL--ERGWNDIGYSFGIGGDGMIYTGRGFNVIGAHAPKY-NDK
Drme_PGRP-LF-RA_N220        KTIQAFHMK--SFGWVDIGYNFLVGGDGQIYVGRGWHIQGQHVNGY-GAI
Trca_PGRP3_partial          QSMQDYHVG--NLKSPDIGYNFVIGGDGNAYVGRGWDIRNFHMD-----D
Drme_PGRP-LD-i1             HKLERSHVG--ELP-----YNFLVAGDCQVFEAQGWHYRSQYPRDLNGID
Apme_PGRP2                  RFAQTFHIE--SRNWSDIGYNFLVGGDGYVYVGRSWDYMGAHAFGY-NNI
Bomo_PGRP4_partial          RLIQTFHIE--SRKWHDIGYNFLVGGDGSAYCGRGWDSVGAHTLGY-NNF
Trca_PGRP2_partial          RFIQTFHIE--SRSWWDIGYNFLVGGDGEAYEGRGWKSEGAHTYGY-NAK
Anga_PGRPLC3                KLIQEFHSSPDSRNFSDIAYQFLVGGDGNAYEGRGWTKQGAHTKGF-NVD
Drme_PGRP-LC-RA             NVVQSFHMD--SWGWDHIGYNFLVGGDGRVYEGRGWDYVGAHTKGY-NRG
Drme_PGRP-LE-RA             RDMQCFHIE--SRGWNDIAYNFLVGCDGNIYEGRGWKTVGAHTLGY-NRI
Drme_PGRP-LA-RE             RTIQDSAIA--EKGLPDIQSNFYVSEEGNIYVGRGWD----WANTY-ANQ
Anga_PGRPcan7_partial       RTLQDAAIA--EKSLQDIPSNFYVGGDGNVYVGRGWD----TANAY-ANM
Anga_PGRPLA_partial         RMLQDAAIG--ERNLPDIPSNFYLGGDGNVYVGRGWD----IANSY-HNR
Bomo_PGRP1                  RNIQTNHME--ALQYWDIGPSFLVGGNGKVYEGSGWLHVGAHTYGY-NSR
Bomo_PGRP2                  NSLRQHHMR--LAGFKDLGYSFVAGGNGKIYEGAGWNHIGAHTLHY-NNI
Bomo_PGRP3                  RSMQKYHN---SLGWGDIGYHFCVGGDGVAYEGRGWNVIGIHAGPA-NKL

Bomo_PGRP6_partial          SLGIGFLGDYREELDPHTRVTDLQIARTKILLEDGV-KRGFLHPK-YYIN
Bomo_PGRP9_partial          SIGILLIGDFIA-----NQPPQAQLQSVKDLIEAGV-RLGHIRSD-YKLI
Bomo_PGRP5_partial          SITIMFLGDYRTD-----KTTPAQFEHLDILLNQLV-KQGVLRPD-YTIL
Bomo_PGRP7_partial          SILIMFLGDYTKD-----KTTPVQFEHLNIVLDQLV-KQGVLRPD-YTLY
Trca_PGRP1_partial          SLGLGFIGNYTSRL-----PNKKQIQAAKDFLQCGV-ELGELGKS-FKLF
Trca_PGRP4_partial          SISVSFIGNFILDE-----LNLNMIDAFQQLMIDGV-NGEKLSKK-YKIM
Bomo_PGRP12_partial         SIGISFIGDYR-----CKVPSVEQIAAAKSLIATGV-ELGFVKPA-HKII
Trca_PGRP5_partial          SIGICVIGDWTKEL-----PPENQLNTVHKLIAFGV-EKGYIRED-YKLL
Trca_PGRP6_partial          SIGICVIGNFQSEL-----PTQTQLDALKQLISCAQ-EGNYVQSD-YRLI
Trca_PGRP7_partial          SIGICFIGCYIQNL-----PPSVALRKAKELIRYGV-KIGAISED-YTLL
Trca_PGRP8_partial          TVTVAFLGNNHFILGLTQQKILLDELDAKAPTFRQA-EAAKMFLE-VAVT
Apme_PGRP1                  SIGICIIGNFVGHT-----PNAAAIEATKNLISYGV-AIGKIQSN-YTLL
Apme_PGRP3_partial          SIGICTIGDFVDRL-----PNNAALKTLEALIKYGI-SLGKISQD-YHII
Apme_PGRP4_partial          SISIAFIGNFLDKS-----ASNKMLNAAHKLILCGK-SKGILRED-VRVI
Hodi_PGRP3                  SLLIGFIGDYEINR-----PSLKQLEAGKQLIECAV-ERGEIEQD-YKLV
Hodi_PGRP2                  SLGIGFIGDFQTNL-----PSSKQLDAGKKFLECAV-EKGEIEDT-YKLI
Hodi_PGRP1                  SIGIAFIGDFTNKL-----PSREMLDAAKDLIVCAI-ELGELTRG-YKLL
Anga_PGRPS3                 SVGMGVIGTFTNGI-----PNAAARNAAQQLISCGV-SLGHIASN-YWLI
Anga_PGRPS2                 SVGMGVIGTFTNAI-----PNAAARTAARNLITCGV-SLGHIASN-YWLI
Drme_PGRP-SA-RA             GTGIAFIGNFVDKL-----PSDAALQAAKDLLACGV-QQGELSED-YALI
Drme_PGRP-SC2-RA            SIGISFLGNYNTNT-----LTSAQITAAKGLLSDAV-SRGQIVSG-YILY
```

```
Drme_PGRP-SC1a-RA        SIGISFLGNYNWDT-----LEPNMISAAQQLLNDAV-NRGQLSSG-YILY
Drme_PGRP-LF-RA_221C     ELVVAFIGPSSSNK-----------KIALELIKQGI-KLGHISKN-YSLI
Drme_PGRP-SD-RA          SLGIAFIGNFEERA-----PNKEALDAAKELLEQAV-KQAQLVEG-YKLL
Drme_PGRP-SC1b-RA        SIGISFLGNYNWDT-----LEPNMISAAQQLLNDAV-NRGQLSSG-YILY
Drme_PGRP-SB1-RA         SIGIVFIGNFERSA-----PSAQMLQNAKDLIELAK-QRGYLKDN-YTLF
Drme_PGRP-SB2-RA         SIGIAFIGNFQTGL-----PPSQMLQAARTLIQIAV-QRRQVSPN-YSVV
Anga_PGRPLB              SVGICLIGDWVADL-----PPKNMLTAAQNLIEYGV-RNGLIAQN-YTLL
Drme_PGRP-LB-RC          SVGIVLIGDWRTEL-----PPKQMLDAAKNLIAFGV-FKGYIDPA-YKLL
Drme_PGRP-LF-RA_N220     SVSIAFIGTFVNME-----PPARQIEAAKRLMDEGV-RLHRLQPD-YHIY
Trca_PGRP3_partial       SIGISFIGNFLHDH-----LTTEMISVAKKLLDEGV-KSGKLARD-YKLV
Drme_PGRP-LD-i1          SLVMAFVGNFSGRP-----PIDCQLMAAQALILESL-KRRILQPI-YQLF
Apme_PGRP2               SIGISFIGTFNTVK-----PSKQQLYVVQKLIELGV-EKGKIAPD-YKLL
Bomo_PGRP4_partial       AIGISFIGTFNNND-----PPKEQLEACRKLIKRGV-DLGKIAKD-YKLF
Trca_PGRP2_partial       SIGIAFIGTFNSFK-----PPERQITACKQLIAKGV-ELGFIRKD-YKLL
Anga_PGRPLC3             SICIAFIGTFIADP-----PPIAQLSAAQQLILLGM-KENYLASN-YSLY
Drme_PGRP-LC-RA          SIGISFIGTFTTRK-----PNERQLEACQLLLQEGV-RLKKLTTN-YRLY
Drme_PGRP-LE-RA          SLGISFIGCFMKEL-----PTADALNMCRNLLARGV-EDGHISTD-YRLI
Drme_PGRP-LA-RE          TLAITFMGDYGRFK-----PGPKQLEGVQFLLAHAV-ANRNIDVD-YKLV
Anga_PGRPcan7_partial    SLAVCFMGDYGRYE-----PNDLQLSALDHLLTFGE-KHRLLTED-YKIV
Anga_PGRPLA_partial      TLSVCFIGDFQTYE-----PKESQFSALHHLLTYGV-IQRKLASD-YKLV
Bomo_PGRP1               SIGVAFIGNFNTDE-----PSGAMLEALRSLLRCGV-ERGHLAGD-YRAV
Bomo_PGRP2               SIGIGFIGDFREKL-----PTQQALQAVQDFLACGV-ENNLLTED-YHVV
Bomo_PGRP3               SIGICLIGDWRVET-----PPAEQLATTKKLLSTGV-EMGAISSD-YKLI

Bomo_PGRP6_partial       GACDFQ-STASPGSNLYKALKSFEHF
Bomo_PGRP9_partial       GHRQVT-PTECPGQRLFDEISKWDHF
Bomo_PGRP5_partial       GQCQVKPLTVSPGRNILKELKDFQHW
Bomo_PGRP7_partial       GQCQVASLTISPGPNVIRELHNLKHW
Trca_PGRP1_partial       GARQVS-ATESPGLKLYRELQNWPHF
Trca_PGRP4_partial       CHNQTK-ATLSPGKNVYKIVKTWPHF
Bomo_PGRP12_partial      GHRQVR-RTACPGDALYELIKTWDKY
Trca_PGRP5_partial       GHRQVR-DTECPGDRLFEEISTWEHF
Trca_PGRP6_partial       GHRQGS-RTSCPGNQLFNEIGGWTHF
Trca_PGRP7_partial       GHCQCR-STESPGRRLFEEIKSWERW
Trca_PGRP8_partial       EGKLER--------------------
Apme_PGRP1               GHRQTT-RTSCPGDSLYELIKTWPHW
Apme_PGRP3_partial       GHRQTK-NTLCPGDKFYEYVQKFPRW
Apme_PGRP4_partial       GGKQVI-ATLSPGFELYKQIQNWPEW
Hodi_PGRP3               GARTIR-QTNSPGKYLFRELQSWKGF
Hodi_PGRP2               GARTVR-PTDSPGTLLFREIQTWRGF
Hodi_PGRP1               GARNVK-ATKSPGDKLYREIQNWEGF
Anga_PGRPS3              GHRQAV-ATACPGNAFFNEIRNWPRF
Anga_PGRPS2              GHRQAV-ATACPGNAFFNEIRTWPRF
Drme_PGRP-SA-RA          AGSQVI-STQSPGLTLYNEIQEWPHW
Drme_PGRP-SC2-RA         GHRQVG-STECPGTNIWNEIRTWSNW
Drme_PGRP-SC1a-RA        GHRQVS-ATECPGTHIWNEIRGWSHW
Drme_PGRP-LF-RA_221C     DDLEKS--------------------
Drme_PGRP-SD-RA          GHRQVS-ATKSPGEALYALIQQWPNW
Drme_PGRP-SC1b-RA        GHRQVS-ATECPGTHIWNEIRGWSHW
Drme_PGRP-SB1-RA         GHRQTK-ATSCPGDALYNEIKTWPHW
Drme_PGRP-SB2-RA         GHCQTK-ATACPGIHLLNELKKWPNW
Anga_PGRPLB              GHRQVR-TTECPGDRLFEEIKTWPHF
Drme_PGRP-LB-RC          GHRQVR-DTECPGGRLFAEISSWPHF
Drme_PGRP-LF-RA_N220     AHRQLS-PTESPGQKLFELMQNWPRF
Trca_PGRP3_partial       AHNQTF-RTESPGPNVYKEIKNWPHF
Drme_PGRP-LD-i1          VLGSYT-------DALQRELRHWPHY
Apme_PGRP2               GHRQVS-QTVSPGDALYSVIQTWPHW
```

```
Bomo_PGRP4_partial          GHRQLS-STLSPGDKLFEIIVEWPHF
Trca_PGRP2_partial          AHRQLE-TTQSPGAALYEEMKTWEHW
Anga_PGRPLC3                GHRQLA-PFESPGKALFDIIKTWPHW
Drme_PGRP-LC-RA             GHRQLS-ATESPGEELYKIIKKWPHW
Drme_PGRP-LE-RA             CHCQCN-STESPGRRLYEEIQTWPHF
Drme_PGRP-LA-RE             AQNQTK-VTRSPGAYVYQEIRNWPHF
Anga_PGRPcan7_partial       AHRQV---------------------
Anga_PGRPLA_partial         ARRQVRG-------------------
Bomo_PGRP1                  AHRQLI-ASESPGRKLYNQIRRWPEW
Bomo_PGRP2                  GHQQLI-NTLSPGAVLQSEIESWPHW
Bomo_PGRP3                  GHNQAM-TTECPGGALLEEISTWDNY
```
