# Supplementary figures and images for: Evolutionary origin of peptidoglycan recognition proteins in vertebrate innate immune system
Source: BMC Evol Biol. 2011 Mar 25;11:79. doi: 10.1186/1471-2148-11-79 (PMC3071341; doi:10.1186/1471-2148-11-79)

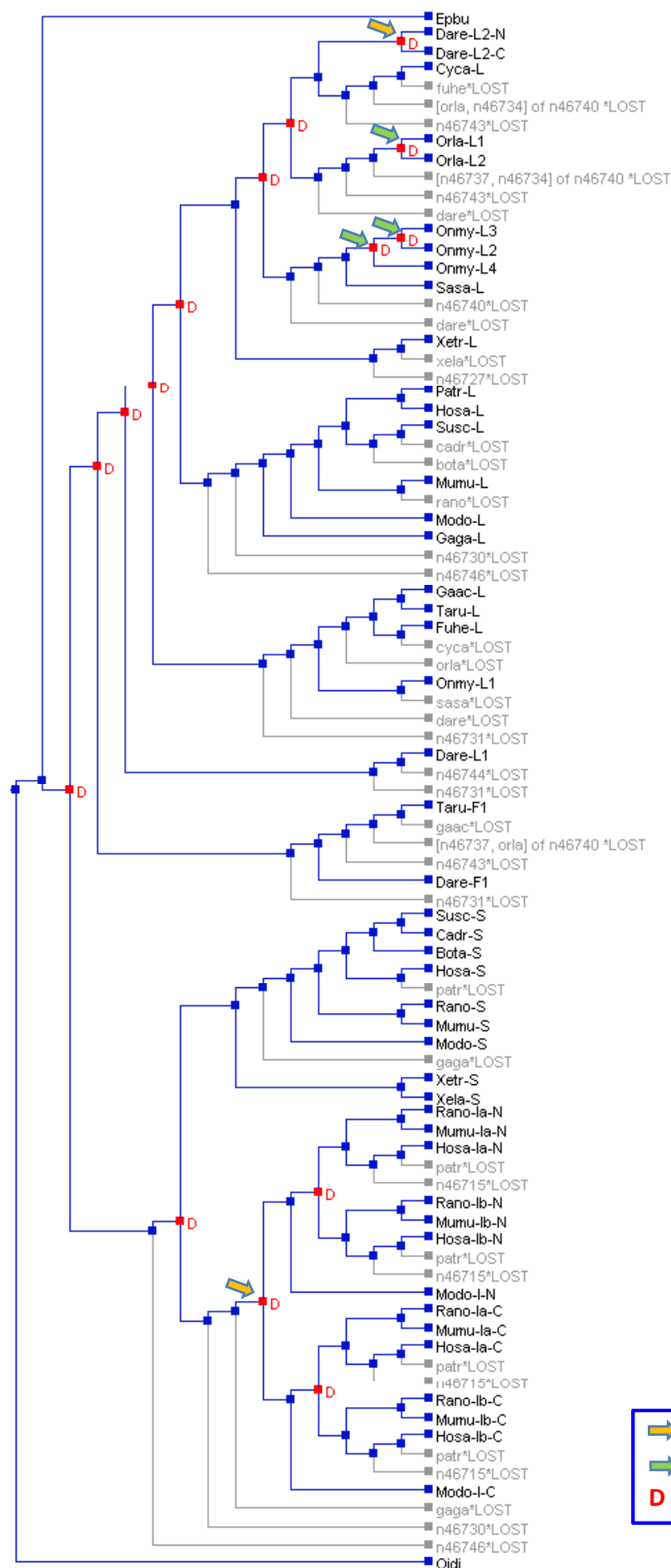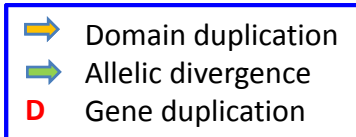

Supplement: Additional file 5 — Reconciled gene tree and species tree of vertebrate PGRPs. NOTUNG analysis predicted 16 duplications and 42 losses. Two of the duplication events are domain duplications and three duplication events are possibly due to allelic divergence. D/L score = 66 [25]. [file 1471-2148-11-79-S5.PDF]

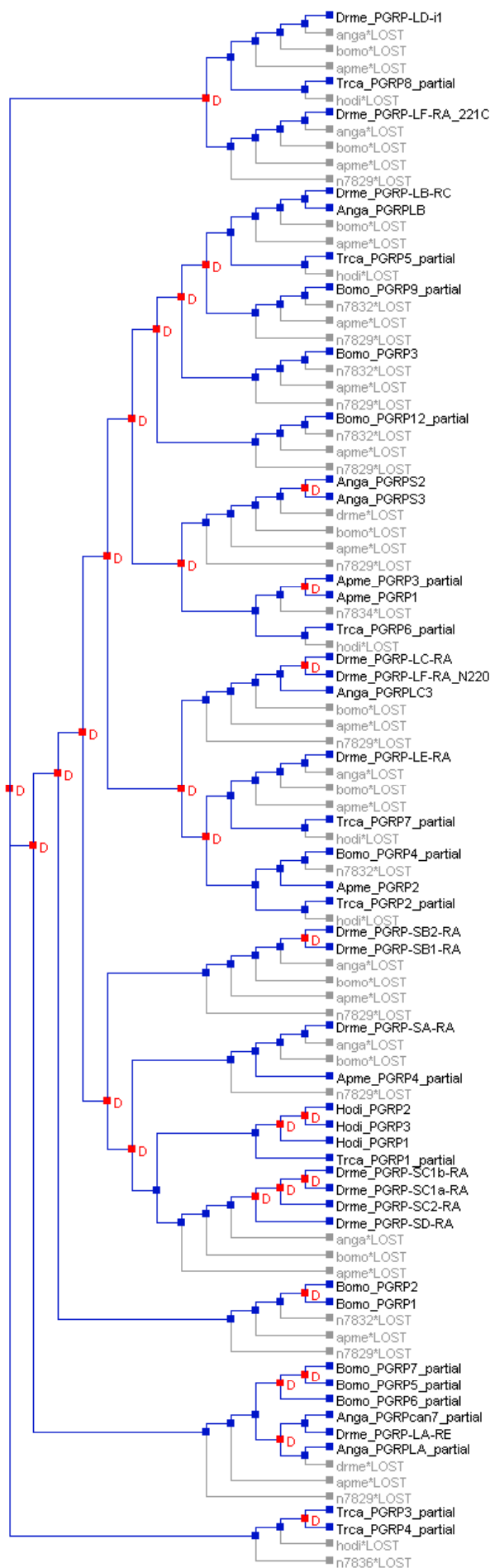

**D** Gene duplication

Supplement: Additional file 6 — Reconciled gene tree and species tree of invertebrate PGRPs. NOTUNG analysis predicted 30 duplications and 53 losses. D/L score = 98 [25]. [file 1471-2148-11-79-S6.PDF]

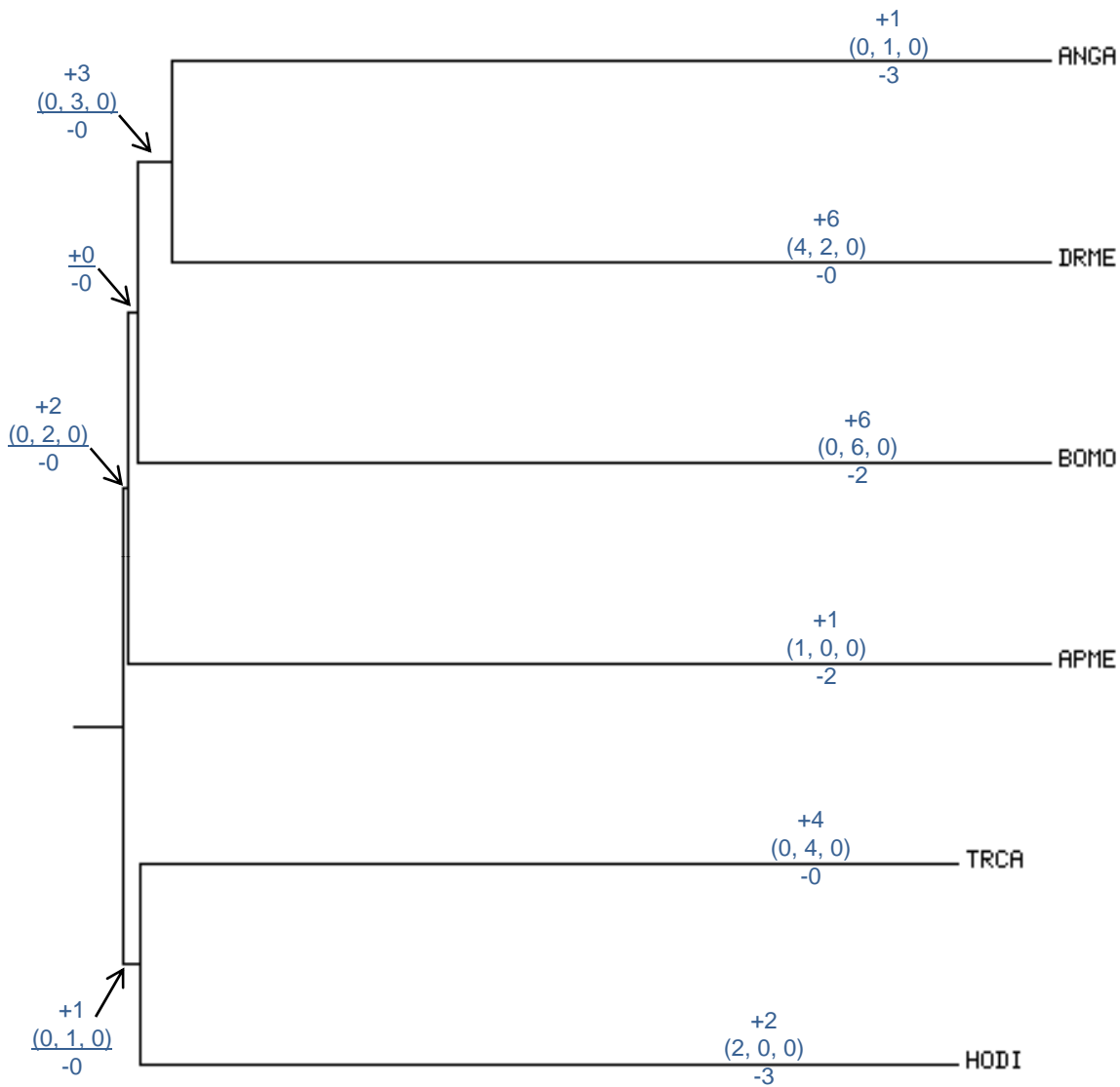

Supplement: Additional file 8 — Average orthologs divergence tree of invertebrate PGRPs. The EvolMAP analysis predicted 26 gains and 8 losses. In-paralogs, and diverged in-paralogs gains constituted 27%, and 73% of total gains, respectively. Gene gains (+) and gene losses (-) are depicted for each branch. Number of in-paralogs, diverged in-paralogs and ambiguous gains are indicated below or next to each gene gain [26]. [file 1471-2148-11-79-S8.PDF]
