# Supplementary material for: Evolutionary origin of peptidoglycan recognition proteins in vertebrate innate immune system
Source: BMC Evol Biol. 2011 Mar 25;11:79. doi: 10.1186/1471-2148-11-79 (PMC3071341; doi:10.1186/1471-2148-11-79)
Supplement: Additional file 9 — Alignment of PGRP ancestral sequences. Alignment of ancestral sequences of PGRP-L and PGRP-S. A dash means the same amino acid as the above. A blue star indicates the amino acid position responsible for Zn2+ ligand binding, whereas a red star indicates the amino acid position responsible for amidase activity. These sites are inferred from the sequences of T7 lysozyme of bacteriophage. [file 1471-2148-11-79-S9.PDF]

```
ancestral_PGRPS      SRSQWGAVPSKCRAKLSLPVRYVIIHHTAGPSCSSLSSCKAQLRSIQRFHMNDRGWDDIGYNFL
ancestral_PGRPL      P-C----A-YRGSPT----LSFLY---TYE---T-F-Q-A-NM--M----QD---------S-V

ancestral_PGRPS      VGSDGHVYEGRGWNIVGAHAKGYNSNSIGISFMGNFTNRAPSAANVRNLLHGVRLRPNYVLKGH
ancestral_PGRPL      -----Y--------W----TR-H--VGY-V--I-DY-STL--S----D--RA---T---TIH--

ancestral_PGRPS      RDVASTECPGDNLYNVIPHF
ancestral_PGRPL      -Q-V--S----A--RE-E--
```
