# Supplementary material for: Evolutionary origin of peptidoglycan recognition proteins in vertebrate innate immune system
Source: BMC Evol Biol. 2011 Mar 25;11:79. doi: 10.1186/1471-2148-11-79 (PMC3071341; doi:10.1186/1471-2148-11-79)
Supplement: Additional file 7 — Average orthologs divergence tree of vertebrate PGRPs. The EvolMAP analysis predicted 14 gains and 14 losses. In-paralogs, diverged in-paralogs and ambiguous gains constituted 36%, 57% and 7% of total gains, respectively. Gene gains (+) and gene losses (-) are depicted for each branch. Number of in-paralogs, diverged in-paralogs and ambiguous gains are indicated below or next to each gene gain [26]. [file 1471-2148-11-79-S7.PDF]

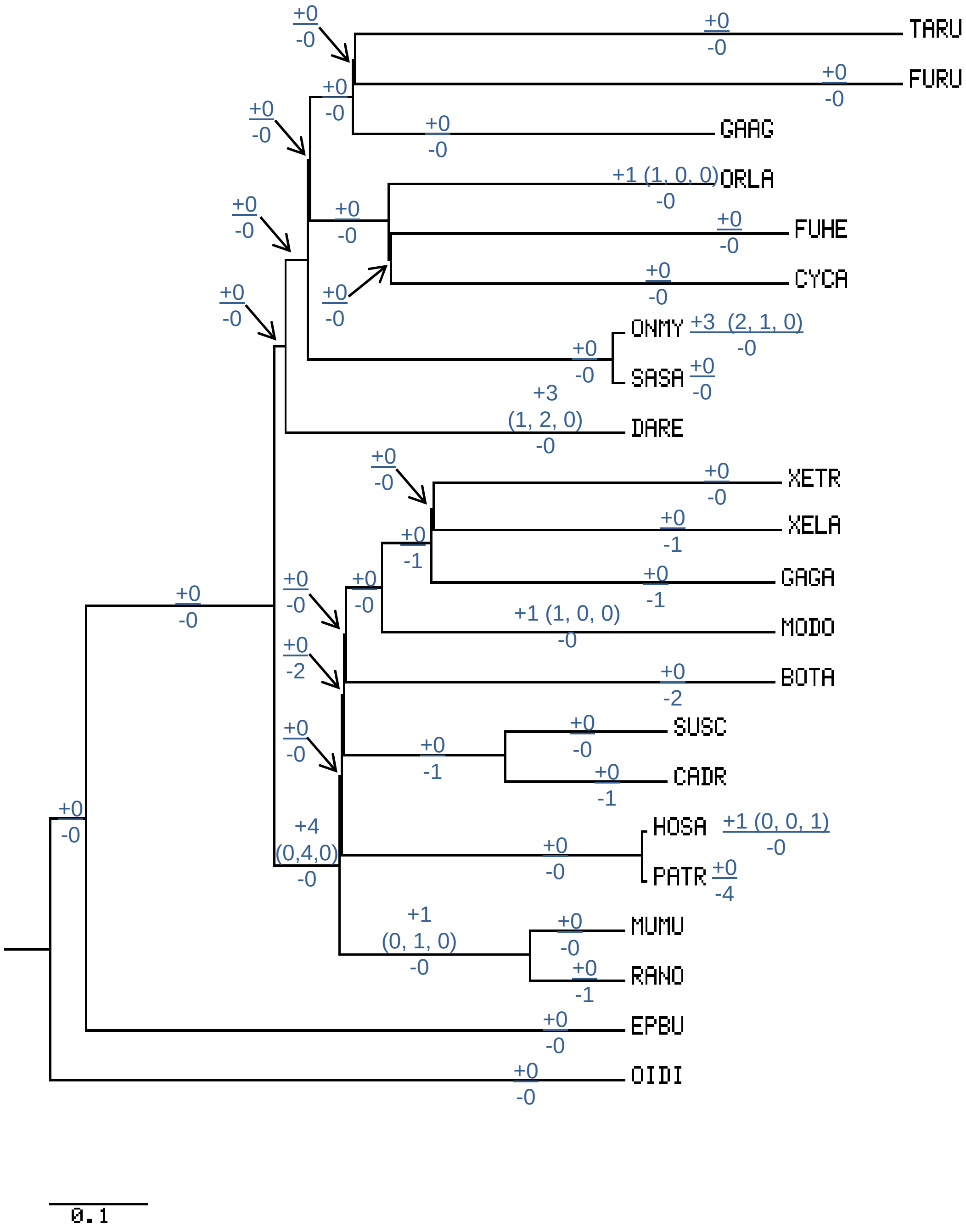

TARU
FURU
GAAG
ORLA
+1 (1, 0, 0)
FUHE
CYCA
ONMY
+3 (2, 1, 0)
SASA
DARE
+3
(1, 2, 0)
XETR
XELA
GAGA
MODO
+1 (1, 0, 0)
BOTA
SUSC
CADR
HOSA
+1 (0, 0, 1)
PATR
+4
(0,4,0)
MUMU
RANO
+1
(0, 1, 0)
EPBU
OIDI
0.1
